# Zinc eluted from glassware is a risk factor for embryo development in human and animal assisted reproduction^†^

**DOI:** 10.1093/biolre/ioaf050

**Published:** 2025-04-02

**Authors:** Tatsuma Yao, Hisato Kobayashi, Tatsuki Hirai, Yuta Tokuoka, Mikiko Tokoro, Yuta Asayama, Yuka Suzuki, Yu Hatano, Hiroki Ikeda, Satoshi Sugimura, Takuya Yamamoto, Takahiro G Yamada, Yoshihiko Hosoi, Akira Funahashi, Noritaka Fukunaga, Yoshimasa Asada, Kazuki Kurimoto, Kazuo Yamagata

**Affiliations:** Research and Development Center, Fuso Pharmaceutical Industries, Ltd., Joto-ku, Osaka 536-8523, Japan; Graduate School of Biology-Oriented Science and Technology, Kindai University, Kinokawa, Wakayama 649-6493, Japan; Department of Embryology, Nara Medical University, Kashihara, Nara 634-0813, Japan; Research and Development Center, Fuso Pharmaceutical Industries, Ltd., Joto-ku, Osaka 536-8523, Japan; Graduate School of Biology-Oriented Science and Technology, Kindai University, Kinokawa, Wakayama 649-6493, Japan; Center for Biosciences and Informatics, Graduate School of Fundamental Science and Technology, Keio University, Yokohama, Kanagawa 223-8522, Japan; Graduate School of Biology-Oriented Science and Technology, Kindai University, Kinokawa, Wakayama 649-6493, Japan; Asada Institute for Reproductive Medicine, Asada Ladies Clinic, Nagoya, Aichi 450-0002, Japan; Research and Development Center, Fuso Pharmaceutical Industries, Ltd., Joto-ku, Osaka 536-8523, Japan; Graduate School of Biology-Oriented Science and Technology, Kindai University, Kinokawa, Wakayama 649-6493, Japan; Graduate School of Biology-Oriented Science and Technology, Kindai University, Kinokawa, Wakayama 649-6493, Japan; Department of Embryology, Nara Medical University, Kashihara, Nara 634-0813, Japan; Department of Biological Production, Tokyo University of Agriculture and Technology, Fuchu, Tokyo 183-8509, Japan; Center for iPS Cell Research and Application, Kyoto University, Sakyo-ku, Kyoto 606-8507, Japan; Institute for the Advanced Study of Human Biology (ASHBi), Kyoto University, Sakyo-ku, Kyoto 606-8501, Japan; Medical-risk Avoidance Based on iPS Cells Team, RIKEN Center for Advanced Intelligence Project, Sakyo-ku, Kyoto 606-8507, Japan; Center for Biosciences and Informatics, Graduate School of Fundamental Science and Technology, Keio University, Yokohama, Kanagawa 223-8522, Japan; Department of Biosciences and Informatics, Keio University, Yokohama, Kanagawa 223-8522, Japan; Graduate School of Biology-Oriented Science and Technology, Kindai University, Kinokawa, Wakayama 649-6493, Japan; Center for Biosciences and Informatics, Graduate School of Fundamental Science and Technology, Keio University, Yokohama, Kanagawa 223-8522, Japan; Department of Biosciences and Informatics, Keio University, Yokohama, Kanagawa 223-8522, Japan; Asada Institute for Reproductive Medicine, Asada Ladies Clinic, Nagoya, Aichi 450-0002, Japan; Asada Institute for Reproductive Medicine, Asada Ladies Clinic, Nagoya, Aichi 450-0002, Japan; Department of Embryology, Nara Medical University, Kashihara, Nara 634-0813, Japan; Advanced Medical Research Center, Nara Medical University, Kashihara, Nara 634-0813, Japan; Graduate School of Biology-Oriented Science and Technology, Kindai University, Kinokawa, Wakayama 649-6493, Japan

**Keywords:** glass, preimplantation embryo development, culture environment, risk factor, zinc

## Abstract

In assisted reproduction, many factors in the culture environment, including light, temperature, pH, and culture media, can reduce preimplantation embryo viability. Laboratory glassware is also a known risk factor for *in vitro* embryos; however, the underlying mechanisms that disrupt embryonic development remain unclear. We identified Zn eluted from glassware as an embryotoxic substance. In mouse embryos, Zn induced delayed development, abnormalities in chromosome segregation, cytokinesis, zygotic gene activation (e.g. *Zscan4a* and murine endogenous retrovirus with leucine, also known as *MERVL*), and aberrantly upregulated developmental gene expression (e.g. *Hoxa1*, *Hoxb9*, *T*, and *Fgf8*) that could be mediated through metal regulatory transcription factors (e.g. *Mtf1*). Subsequently, Zn exposure led to significantly reduced blastocyst formation. Post-implantation, Zn-exposed embryos were associated with normal birth rates, however, the birth weight increased by an average of 18% compared with embryos cultured without Zn. Furthermore, Zn exposure affected the development of bovine and human embryos, with species-based variation in the strength and timing of these effects. To mitigate these embryotoxic effects, we identified a method to prevent glass toxicity using chelating agents. This research not only highlights the importance of risk control in embryo culture but also facilitates the development of safe and effective methods for assisted reproduction.

## Teaser

Zn leached from glassware is a risk factor in ART and should be strictly controlled because it can alter gene expression via metal regulatory transcription factors and disrupt embryonic development.

## Graphical Abstract

**Graphical Abstract:**
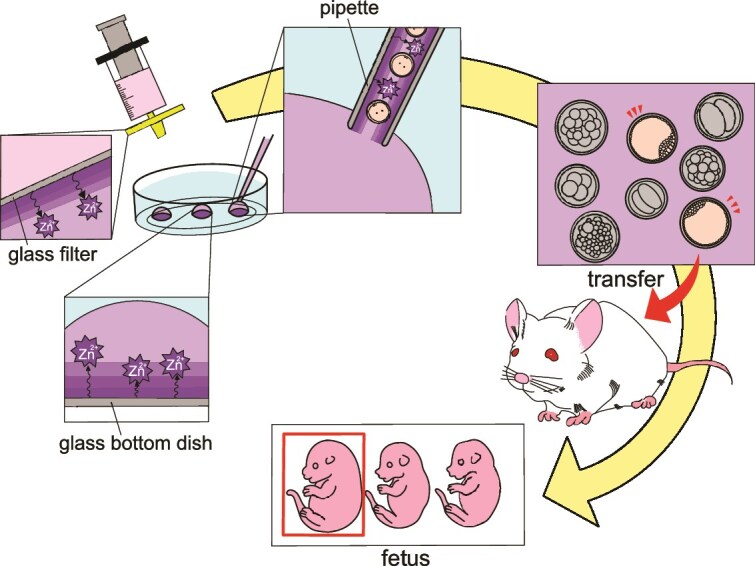

## Introduction

Proper culture technologies for mammalian preimplantation embryos are essential in various fields, including medicine, the agricultural industry, and academic research. Since the successful development of assisted reproductive technology (ART) for human reproduction in 1978, ART has resulted in >9 million births globally, with >2.4 million treatments performed annually [1]. ART is also used in livestock breeding to increase the reproductive efficiency and breeding outcomes beyond artificial insemination. In this field, embryos for transfer are recovered through uterine flushing after *in vivo* development or produced using *in vitro* culture. However, in recent years, the use of *in vitro*-produced embryos in cattle has increased [2]. Embryo culture technology is not only used in developmental biology research but also in the fields of developmental toxicology and engineering, including the production of genetically modified and cloned animals. To improve the success rate of ART and support developmental research, specific culture media, methods, and equipment have been developed with considerable success [3, 4]. However, various risk factors remain that may disrupt preimplantation embryo development. For example, embryo development is suppressed when poor quality mineral oil is used for microdroplet culture [5, 6]. Furthermore, exposure to light and temperature changes that may affect embryo quality cannot be avoided under current culture conditions, and the influence of environmental oxidative stress must also be considered [7–11]. Some risk factors require further investigation. For example, some sterile syringe filters have been suggested to contain embryotoxic substances [12]; however, the causative agents remain unknown. Zn in silicone oil used for microdroplet culture has been shown to migrate into the embryo culture medium and inhibit mouse embryonic development in a concentration-dependent manner [13]; however, why embryonic development rates are reduced, whether post-implantation development is affected, and how embryos from other animals, including humans, are affected by Zn remain unclear. Furthermore, the likelihood of these embryotoxic substances entering the culture medium from other laboratory materials remains unverified. Exploring the source of such risk factors, identifying the underlying causes of toxicity, and controlling them appropriately are crucial to further improve and stabilize ART outcomes and related developmental research.

Laboratory glassware, such as pipettes, is commonly used in embryo culture but has known embryotoxic properties and should be washed before use [14, 15]. However, detailed studies have not been conducted to determine the types of glassware that have adverse effects on embryonic development, the underlying mechanisms, or whether they affect non-mouse mammalian embryos. Therefore, we aimed to identify the embryotoxic substance in glassware and determine when and how it affects preimplantation mouse, bovine, and human embryos. Using morphological evaluation, live-cell imaging, and RNA-seq, we sought to uncover the molecular mechanisms through which the identified toxic substance interferes with embryonic development. Additionally, using birth rate and birth weight as outcomes, we investigated potential methods of counteracting this toxicity. To establish safer and more efficient culture methods, it is important to recognize the degree of embryotoxicity and the causative agent in glassware used for ART.

## Materials and methods

### Ethics statement

All methods were performed in accordance with the relevant guidelines and regulations. This study was conducted in accordance with the ARRIVE guidelines. The animal experiments were approved by the Ethics Committee for the Care and Use of Experimental Animals at Kindai University (permit numbers: KABT-27-004 and KABT-31-016). The experiments with human embryos were approved by the Institutional Review Board of Asada Ladies Clinic (Approval numbers: 2013–002 and 2017–05), Kindai University (approval number: H27–2-008), Osaka University (previous affiliation of the corresponding author (K. Y.); approval number: 25–14), and the Japanese Society of Obstetrics and Gynecology (approval number: 123; date of approval: 04/25/2018). Moreover, informed consent was obtained from all patients before inclusion in the study.

### Animals

C57BL/6N, ICR, and BDF1 mice (male and female, 10–16 weeks old), as well as pseudopregnant ICR female mice on Day 1.5, were obtained from Japan SLC Inc. (Shizuoka, Japan). C57BL/6N mice were used in the embryo culture experiments, unless otherwise stated. All mice were maintained at 23°C and 50% humidity under a 12-h photoperiod, with *ad libitum* access to water and food pellets. Euthanization was performed via cervical dislocation.

### Instruments and reagents

All reagents were purchased from Fujifilm Wako Pure Chemical Corp. (Osaka, Japan), unless otherwise stated. To verify the effects of trace elements (B and Zn) and chelating agent (ethylenediaminetetraacetic acid [EDTA]) on the embryo culture, stock solutions of boric acid (027–02192; Fujifilm), zinc chloride (260–01021; Fujifilm), and EDTA-2Na (343-01861; Dojindo, Kumamoto, Japan) at concentrations of 10, 50, and 10 mM were prepared, respectively. Plastic dishes for embryo culture were obtained from AGC Techno Glass Co., Ltd. (1000–035; Shizuoka, Japan). To evaluate the toxicity of 33-mm diameter syringe filters (0.22 μm pore size) obtained from Merck (SLGP033RS [Millex GP], SLGV033RS [Millex GV]; Darmstadt, Germany), Sartorius (16534 [Minisart], 17823 [Minisart Plus]; Goettingen, Germany), and GE Healthcare (6901–2502 [GD/X]; Buckinghamshire, UK), filtrate collected by sequentially passing 1 ml of KSOM^AA^ medium containing 0.1% bovine serum albumin (BSA) (MR-106D; Merck) was used. To evaluate the toxicity of 14-mm diameter glass-bottom dishes obtained from AGC Techno Glass (3971–035), Matsunami Glass (D11130H; Osaka, Japan), and MatTek (P35G-1.5-14-C; Ashland, MA, USA), polyethylene terephthalate (PET) film (Lumirror T60; Toray Industries, Inc., Tokyo, Japan) was used. To determine whether the toxicity of the glass-bottom dish was caused by the cover glass, embryo culture was carried out with a cover glass (C1200; Matsunami Glass). The toxicity of the glass capillaries obtained from ERMA (Plain; Tokyo, Japan) was evaluated using effluent obtained after aspirating 5 μl of BSA-free KSOM^AA^ (MR-107D; Merck).

### *In vitro* fertilization

Mouse *in vitro* fertilization (IVF) was performed as described previously [16]. In brief, the mice were superovulated via 10 IU intraperitoneal injection of pregnant mare serum gonadotropin (ASKA Pharmaceutical Co., Ltd., Tokyo, Japan), followed 48 h later by 10 IU of human chorionic gonadotropin (ASKA Pharmaceutical Co., Ltd.). Cumulus-oocyte-complexes (COCs) were collected from the oviducts 14–16 h after the injections and added to 200-μl drops of TYH medium [17] under liquid paraffin (26117-45; Nacalai Tesque, Kyoto, Japan) at 37°C and 6% CO_2_ in a humidified atmosphere. Spermatozoa were collected from the cauda epididymis and incubated in 200-μl drops of TYH medium for 1.5 h at 37°C with 6% CO_2_ in a humidified atmosphere. The sperm suspension was added to the COCs suspension at a concentration of 50–100 sperm/μl and incubated for 1.5 h at 37°C and 6% CO_2_ in a humidified atmosphere. After insemination, the cumulus cells were removed using hyaluronidase (Type-IS, 150 U/ml; Sigma-Aldrich, St. Louis, MO, USA) and a plastic pipette (CooperSurgical, Målov, Denmark). Subsequently, fertilized eggs with two pronuclei were selected after 5–6 h of insemination.

Bovine IVF was performed as described previously [18]. In brief, ovaries from Japanese Black or Japanese Black × Holstein breeds were collected from a local slaughterhouse. COCs were aspirated from small follicles (2–6 mm in diameter) and placed in 25 mM HEPES-buffered TCM199 (Gibco M199, 12340-030; ThermoFisher Scientific, Waltham, MA, USA) with 5% new born calf serum (S0750; Biowest, Nuaillé, France) and 0.1 IU/ml recombinant human follicle-stimulating hormone (Follistim; MSD, Tokyo, Japan). Subsequently, the COCs in tubes (20–40 COCs / 500 μl medium under 300 μl liquid paraffin in 1.5 ml plastic tubes) were transferred to a portable incubator (01213900; Fujihira Industry Co., Ltd., Tokyo, Japan) and cultured at 38.5°C during transport. After 22 h of incubation, matured oocytes were inseminated with pre-thawed and washed sperm at a concentration of 3 × 10^3^ sperm/μl in Brackett and Oliphant solution [19] supplemented with 5 mM hypotaurine (H1384; Sigma-Aldrich), 2 U/ml heparin (Novo-Heparin Injection 1000; Aventis Pharma Ltd., Tokyo, Japan), and 10 mg/ml BSA (A7030; Sigma-Aldrich) at 38.5°C and 6% CO_2_ in a humidified atmosphere for 6 h. After insemination, cumulus cells were removed using a plastic pipette and fertilized eggs with polar bodies were selected.

### Embryo culture

All embryo culture dishes were prepared 24 h before embryo culture and equilibrated in a CO_2_ incubator. Subsequently, 10–12 mouse embryos were cultured in 5-μl drops of KSOM^AA^ medium containing 0.1% BSA or BSA-free KSOM^AA^ medium under liquid paraffin at 37.0°C and 6% CO_2_, 5% O_2_, and 89% N_2_ in a humidified atmosphere for 4 days. KSOM^AA^ medium containing 0.1% BSA was used for mouse embryo culture, unless otherwise stated.

Nine to eleven or four bovine embryos were cultured in 50 or 5-μl drops, respectively, of IVD101 medium containing 0.1% BSA (IFP9651; Research Institute for the Functional Peptides Co., Ltd., Yamagata, Japan) or CR1aa medium containing 5% calf serum and 0.3% BSA under liquid paraffin at 38.5°C and 6% CO_2_, 5% O_2_, and 89% N_2_ in a humidified atmosphere for 8 days. IVD101 medium was used for bovine embryo culture, unless otherwise stated.

All human embryos were obtained from four patients who had undergone fertility treatment at the Asada Ladies Clinic. The maternal ages at the time of embryo freezing were 28, 31, 32, and 37 years. Human embryos frozen at ~20 h post-insemination were thawed using thawing media for vitrified embryos (VT506; Kitazato Corp., Shizuoka, Japan) according to the manufacturer’s protocol and randomly allocated to each culture drop after all embryos were mixed. Subsequently, 2–4 human embryos were cultured in 5-μl drops of KSOM^AA^ medium supplemented with 0.1% human serum albumin (9988; Irvine Scientific, Irvine, CA, USA) under liquid paraffin at 37.0°C in a humidified atmosphere with 6% CO_2_, 5% O_2_, and 89% N_2_ for 6 days.

### Live-cell imaging

To visualize chromosomes, messenger RNA (mRNA) of histone H2B-mCherry (mouse, 10 ng/μl; human, 20 ng/μl) was injected into the mouse and human ooplasms using a piezo manipulator (PMM-150FU; Prime Tech Ltd., Ibaraki, Japan). To avoid unexpected toxicity from the cover glass, we used an integrally molded film-bottom dish made of polystyrene (FD20301; Matsunami Glass) instead of the commonly used glass-bottom dish. To prevent movement during imaging, embryos were adhered and arranged in each 2.5-μl droplet of BSA-free KSOM^AA^ medium at the bottom of the dish, and then 2.5 μl of KSOM^AA^ medium containing 0.2% BSA (A3311; Sigma-Aldrich) was added. The embryos were then imaged three-dimensionally using a box-type confocal laser microscope (CV1000; Yokogawa, Tokyo, Japan) under the culture conditions described in the “*Embryo culture*” subsection. Images were captured every 10 min for 4 days (mouse) or every 15 min for 6 days (human). The imaging parameters were set as follows: excitation wavelength, 561 nm; emission wavelength, 671/73 nm; laser power, 0.05 mW; exposure time, 100 ms; gain, 100%; range, 100 μm (mouse) or 120 μm (human); and slices, 51. Image acquisition was performed using CV1000 software (version 1.06, Yokogawa), and time-series montage image creation was performed using Fiji software [20]. Segmentation and counting of nuclei in the three dimension images at each time point were performed using QCANet [21], a deep-learning-based algorithm.

### Immunostaining

Details of the antibodies used are provided in Supplemental Table S1. Zona pellucida of the mouse embryos cultured in the medium supplemented with or without Zn was removed using acidic Tyrode’s solution, and then the samples were fixed in formalin (HT5011; Sigma-Aldrich) for 30 min and permeabilized in phosphate-buffered saline (PBS) containing 0.25% Triton X-100 (T9284; Sigma) for 20 min. After blocking with PBS containing 3% BSA, mouse monoclonal anti-metallothionein and rabbit polyclonal anti-NANOG primary antibodies in PBS containing 3% BSA were added, and the samples were incubated for 2 h. After washing with PBS containing 3% BSA, CF488A goat anti-mouse IgG (H + L) and CF555 anti-rabbit IgG (H + L), the secondary antibodies, in PBS containing 3% BSA were added, and the samples were incubated overnight at 4°C. DNA was stained with 2 μg/ml 4′,6-diamidine-2′-phenylindole dihydrochloride (DAPI; 10236276001; Roche, Indianapolis, IN, USA) in PBS containing 0.02% polyvinyl alcohol (PVA; P8136; Sigma-Aldrich) for 60 min, and fluorescence images were acquired using a CV1000 microscope (Yokogawa). The fluorescence intensities in images of maximum intensity projection were measured using Fiji software after segmentation using Otsu’s algorithm [22].

### Murine embryo transfer

Embryo transfer was performed surgically as described previously [23]. In brief, six to seven morula or blastocyst-stage embryos at ~76 h post-insemination were transferred into each uterus of the pseudopregnant ICR strain mice on Day 2.5 post-coitum. The pregnant mice underwent Cesarean section 18.5 days post-coitum to evaluate the development of full-term neonates, and body and placental weights of the resulting litters were recorded.

### Culture medium component analysis

Multiple 5-μl KSOM^AA^ drops incubated on glass-bottom (MatTek) or plastic dishes (AGC Techno Glass) for 4 days under the conditions described in the “*Embryo culture*” section were collected and subjected to subsequent analysis. Aliquots (5 μl) of the sample were mixed with 15 μl of 6.7% sulfosalicylic acid containing 3.4 mM DL-norvaline (N0304; Tokyo Chemical Industry, Co., LTD., Tokyo, Japan) as an internal standard. After centrifugation at 16 000 × g for 15 min, 3 μl of the supernatant, 12 μl of AccQ Tag Ultra borate buffer (186003836; Waters Corp., Milford, MA, USA) containing 125 mM NaOH, and 6 μl of AccQ•Tag Ultra derivatization reagent (186003836; Waters) were mixed, vortexed for 10 s, incubated for 1 min at 22°C –28°C, and placed in a block heater at 55°C for 10 min. One microliter of the mixture was injected into an Acquity UPLC system (Waters) equipped with an AccQ Tag Ultra column (2.1 × 100 mm, 1.7 μm; Waters) connected to a tunable dual-wavelength UV/Visible detector (Waters). The chromatographic parameters were set as described in the instruction manual (715021297KI; UPLC Amino Acid Analysis Solution System Guide; Waters). For the analysis of the anions, pyruvate, and lactate, a 5-μl aliquot of the sample or standard solution was mixed with 395 μl of 0.08 mM lithium fluoride as an internal standard. The mixtures were filtered for deproteinization using 10 K molecular weight cut-off filters (USY-1; Advantec Toyo Kaisha, Ltd., Tokyo, Japan) washed thrice with ultrapure water. The filtrate (25 μl) was introduced into an ion chromatography (IC) system (Dionex ICS-2100; ThermoFisher Scientific) equipped with an IonPac AS18 column (2 × 250 mm, ThermoFisher Scientific). The chromatographic parameters were set as follows: column temperature, 30°C; detector cell temperature, 30°C; suppressor, anion electrolytically regenerated suppressor in the external mode; flow rate, 0.25 ml/min; eluent, potassium hydrate (KOH) from the eluent generator. The gradient conditions of KOH were as follows: 2 mM, −10.0–7.5 min; 2–40 mM, 7.5–11.5 min; 40 mM, 11.5–16.0 min; 40–50 mM, 16.0–17.0 min; 50 mM, 17.0–23.5 min; 50–99 mM, 23.5–24.5 min; 99 mM, 24.5–31.9 min; 99–39 mM, 31.9–32.9 min; and 39 mM, 32.9–33.0 min. For cation analysis, the sample was pretreated as for anions; however, 0.02 mM strontium chloride was used as an internal standard in addition to lithium fluoride. The filtrate (25 μl) was analyzed using an IC system equipped with an IonPac CS16 column (3 × 250 mm, ThermoFisher Scientific) with the following chromatographic parameters: column temperature, 40°C; detector cell temperature, 35°C; suppressor, cation electrolytically regenerated suppressor in external mode; flow rate, 0.4 ml/min; and eluent, 30 mM methanesulfonic acid (000-48412; Kishida Chemical Co., Ltd., Osaka, Japan). For glucose analysis, a 5-μl aliquot of the sample or standard solution was mixed with 45 μl of 0.5 mM stable isotope-labeled D-glucose (DLM-2062-0.5; Cambridge Isotope Laboratories, Inc., Tewksbury, MA, USA) as an internal standard and 350 μl of acetonitrile. After centrifugation at 16 000 × g for 15 min, 1 μl of the supernatant was injected into an Acquity UPLC system equipped with an Acquity UPLC BEH amide column (2.1 × 50 mm, 1.7 μm; Waters) connected to a 3200 Q TRAP hybrid tandem mass spectrometer (MS/MS) (Sciex, Framingham, MA, USA). The chromatographic parameters were set as follows: column temperature, 65°C; flow rate, 0.15 ml/min; eluent A, 0.1% ammonium hydroxide solution; eluent B, 0.1% ammonium hydroxide acetonitrile; and total eluent = 20% eluent A or 80% eluent B. The parameters for mass spectrometry were set as follows: scan type, multiple reaction monitoring; polarity, negative; curtain gas flow, 25; collision gas, 5; ionspray voltage, −4500; temperature, 350°C; ion source gas 1, 80; ion source gas 2, 80; quadrupole 1, 178.988; quadrupole 3, 89.00; dwell time, 250 ms; declustering potential, −20; entrance potential, −2; collision cell entrance potential, −12; collision energy, −12; and collision cell exit potential, 0. Data acquisition and quantification were performed using Analyst software (Version 1.6.2, Sciex) for glucose and Chromeleon software (Version 6.80 SR13, ThermoFisher Scientific) for other components according to the user manual.

### Trace element analysis

Dish samples were collected using the method for culture medium component analysis. The filter samples were prepared by passing 1 ml of KSOM^AA^ medium through a filter with or without glass fibers (Sartorius). Ultrapure water prepared using a Milli-Q Integral 3 system (Merck) and ultrapure nitric acid (Ultrapur-100; Kanto Chemical Co., Inc., Tokyo, Japan) was used for this analysis. All plastic containers, tubes, and pipettes were prewashed with 5% nitric acid solution. A 0.1-ml aliquot of the sample or standard solution was mixed with 3.9 ml of 5% nitric acid solution and incubated for 20–24 h at 22°C–28°C. After centrifuging the sample at 400 × g for 10 min, the supernatant was collected and introduced into an inductively coupled plasma mass spectrometry (ICP-MS) system (7900; Agilent). All measurements were performed in He mode with the following operating parameters: RF power, 1550 W; plasma gas flow, 15 L/min; carrier gas flow, 1.05 L/min; helium gas flow, 4.3 ml/min; octupole bias, −18.0 V; energy discrimination, 5.0 V; and sampling depth, 8.0 mm. ICP-MS optimization and semi-quantification of elements were performed using a tuning solution for ICP-MS (5185–5959; Agilent) and quantification of Zn was performed using Zn standard solution for ICP-MS (260–02241; Fujifilm Wako Pure Chemical). Data acquisition and quantification were performed using MassHunter software (Version 4.2, Agilent).

### Preparation of the cDNA and RNA-sequencing libraries

Embryos at the two-cell stage (n = 20) or blastocyst stage (n = 8) were pooled at the bottom of a 0.5-ml tube (N8010737; Applied Biosystems, Waltham, MA, USA) using a mouth pipette with a very small volume of washing medium (BSA-free HEPES-buffered KSOM^AA^ containing 0.1% PVA) and snap-frozen in liquid nitrogen. Total RNA was extracted using an RNeasy Micro Kit (74004; Qiagen, Hilden, Germany) according to the manufacturer’s protocol. One nanogram of total RNA was quantified using a Qubit RNA HS Assay Kit (Q32852; ThermoFisher Scientific) and was used for cDNA synthesis and library preparation using a SMART-Seq HT Plus Kit (R400749; Takara Bio, Shiga, Japan) with 11 cycles of PCR. RNA-seq libraries were quantified with real-time PCR using a KAPA Library Quantification Kit (Kapa Biosystems, Woburn, MA, USA).

### Next-generation sequencing and data processing

All RNA-seq libraries were mixed and used for single-end 76-bp sequencing on the NextSeq 500 system (Illumina, San Diego, CA, USA) using a High Output Kit v2. Base-calling was performed using NextSeq 500/550 RTA software (v. 2.11.3). FASTQ files were generated using the bcl2fastq tool (v. 2.20.0.422). Using RaNA-seq, an open bioinformatics tool for RNA-seq (https://ranaseq.eu/) [24], transcripts per million (TPM) values were calculated for mouse genome assembly, and the annotation of single-copy genes was performed in Ensembl (mm10; 34 866 genes). For expression analysis of transposable elements (TEs), the FASTQ data were trimmed to remove adapter sequences and reads of poor quality using fastp (v. 0.20.0) and were aligned to mm10 mouse genome assembly using the STAR tool (v. 2.6.0). The best-matched reads on the annotated TE loci were counted using HTSeq 0.10.0.

### Transcriptome analysis

For the gene expression analyses, TPM + 1 values were converted to log_2_, and genes satisfying the following criteria were subjected to subsequent procedures: log_2_ TPM + 1 > 2 in at least one sample; largest difference in log_2_ TPM + 1 between individual samples >1 (13 689 genes). Principal component analysis (PCA) and hierarchical clustering were performed using the prcomp and hclust functions, respectively, in the R program suite [25] with the default settings. Differentially expressed genes between control and Zn-treated embryos were defined using the following criteria: difference in average log_2_ TPM + 1 > 1, *P*-value (*t*-test) <0.01, and *q* values (Benjamini-Hochberg) <0.1. Gene ontology analysis was performed using DAVID [26, 27] and the Gene Ontology (GO) Terms Classification Counter [28]. To compare the gene expression data with previously reported transcriptional dynamics of mouse preimplantation development [29], FASTQ files were downloaded and TPM values were calculated using the RaNA-seq tool. The TPM values of the TEs were calculated, and the differentially expressed TEs were identified using DESeq2 (v. 1.28.1) (Padj <0.01). Moreover, a volcano plot was generated by plotting the log_2_FoldChange and Padj values. Enrichment of transcription factor–binding motifs was calculated for genomic sequences of differentially expressed genes (transcription start site ±1 kb) using findMotif in the HOMER-4.10 tool [30].

### Statistical analyses

Statistical analyses were performed using R (version 4.3.1). Two-tailed Fisher’s exact tests were used to compare categorical variables, with Holm adjustment of *P*-values for multiple comparisons. Shapiro–Wilk tests were used to confirm normality. The F-test and Bartlett’s test were used to confirm the variability. Depending on the number of groups, the normality, and the variability of the dataset, either the two-tailed Student’s *t*-test, Welch’s *t*-test, Wilcoxon rank-sum test, Steel test, or Steel–Dwass test was used to compare continuous variables. The source codes for the Steel and Steel–Dwass tests for R were obtained from EZR software (Version 1.54) [31]. Details of the replicates and the statistical tests for each dataset are provided in the figure legends. A *P*–value of <0.05 was considered significant, unless otherwise stated.

## Results

### Glass toxicity in preimplantation mouse embryos

**Figure 1:**
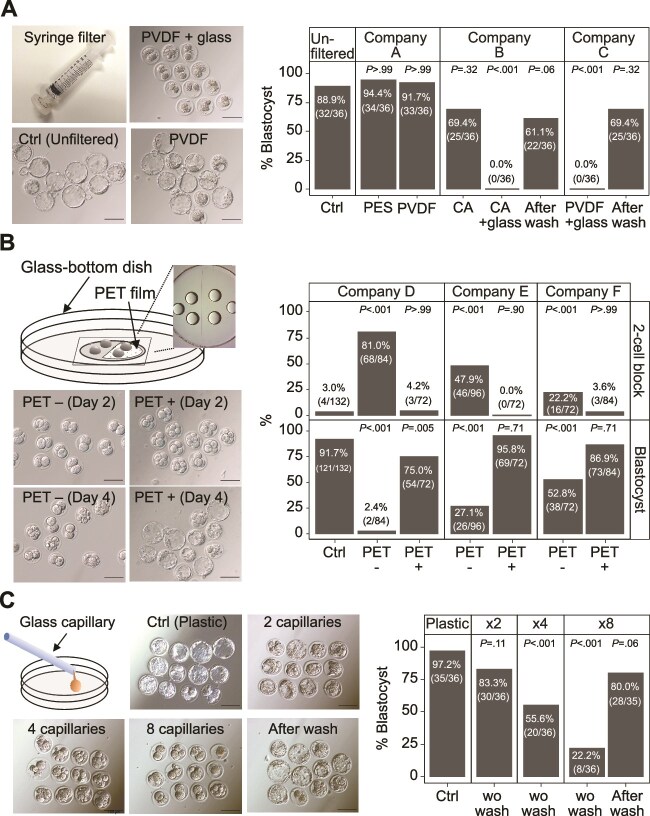
Glass toxicity in preimplantation mouse embryos. Pronuclear-stage embryos were cultured for 4 days with or without glass contact. (A) Comparison of filtrate collected by passing 1 ml of medium through a syringe filter with or without glass fibers and a syringe filter with glass fibers after washing. Scale bars indicate 100 μm. Right column: blastocyst formation rate on Day 4. Ctrl indicates culture medium in a syringe rinsed with 1-ml of medium; PVDF, polyvinylidene difluoride; PES, polyethersulfone; CA, cellulose acetate. The experiments were replicated thrice independently. (B) Comparison of the culture medium on PET film (PET+) and on cover glass (PET−) in a glass-bottom dish. Scale bars indicate 100 μm. Right column: two-cell block rate and blastocyst formation rate on Days 2 and 4. PET, polyethylene terephthalate; two-cell block, embryos at the two-cell stage until 48 h post-insemination; Ctrl, culture medium on plastic dish. The experiments were replicated four times independently. (C) Comparison of the number of unwashed or washed glass capillaries used to aspirate and drain 5 μl of BSA-free medium. Scale bars indicate 100 μm. Right column: blastocyst formation rate on Day 4. Ctrl indicates 5 μl of BSA-free medium aspirated and drained with an unused plastic capillary. The experiments were replicated thrice independently. *P-*values as compared to Ctrl were calculated by two-tailed Fisher’s exact tests and adjusted using the Holm procedure.

Generally, culture media are sterilized using filters after preparation [12]. We filtered KSOM^AA^ medium [32] using filters from different companies before culturing fertilized mouse eggs and found that some products were associated with significantly lower blastocyst formation rates than others (Figure 1A). This effect was eliminated when the filters were washed with the culture medium before use (Figure 1A, Supplemental Figure S8C). A common feature associated with filters exhibiting negative effects on blastocyst formation was the presence of glass fibers in the filter.

Subsequently, we examined the effect of culturing embryos in glass-bottom dishes. We observed developmental arrest at the two-cell stage (two-cell block) and inhibition of embryonic development up to the blastocyst stage in all three types of glass-bottom dishes used (Figure 1B). When the contact area between the cover glass and the culture drop was replaced with a constant volume of culture medium, the embryo developmental rate decreased in an area-dependent manner (Supplemental Figure S1A, B). The addition of a PET film to the surface of the glass to avoid direct contact with the medium resulted in a blastocyst rate comparable to that in the control dish, even in the most toxic glass-bottom dish (Figure 1B).

Next, we examined the toxicity of the glass capillaries used for the manipulation of embryos and found that the use of these pipettes negatively affected embryonic development in a number-dependent manner (Figure 1C). These results indicated that some glass products are toxic and inhibit embryonic development.

### Trace Zn as an inhibitor of preimplantation development *in vitro*

**Figure 2:**
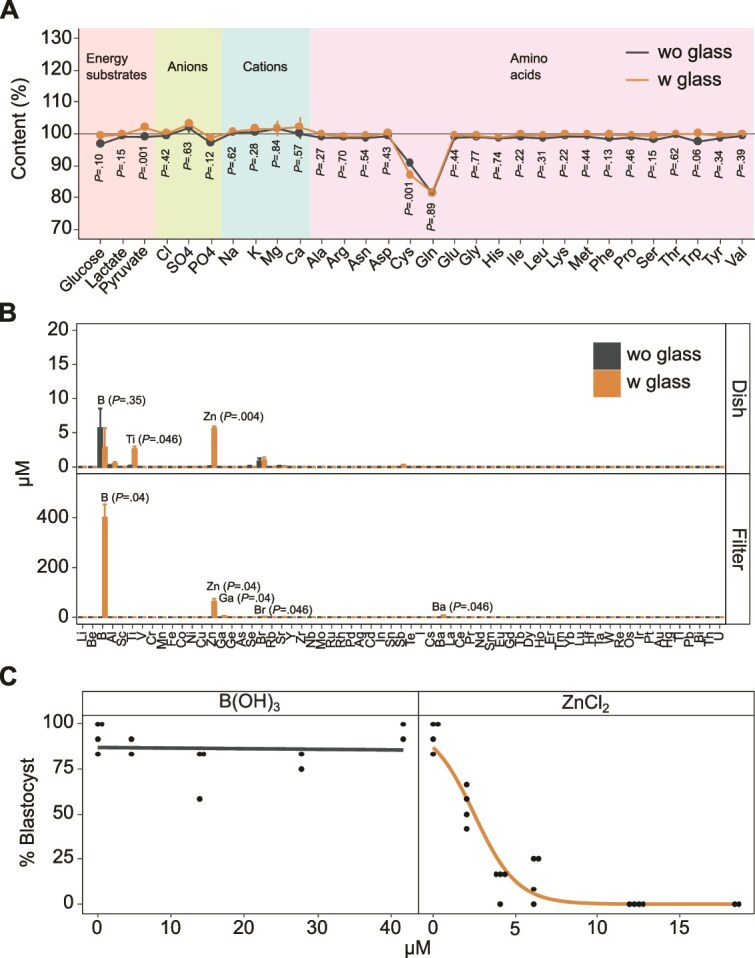
Trace elements in the culture medium exposed to glass and their effects on preimplantation mouse embryos. (A) Content calculated by dividing the concentration of components in the culture medium on plastic (wo glass) or glass (w glass) in glass-bottom dishes after 4 days of culture by the concentration before incubation. Data are presented as the mean ± SEM. The experiments were replicated five times independently. *P*-values were calculated by the two-tailed Wilcoxon rank-sum test for Ile, Lys, Cl, and Mg, two-tailed Welch’s *t*-test for Trp and two-tailed Student’s *t*-test for other components. (B) Concentration of trace elements in the culture medium on plastic (wo glass) or glass (w glass) in glass-bottom dishes after 4 days of culture (top) or passed through a syringe filter with or without glass (bottom). Data are presented as the mean ± SEM. The experiments were replicated three times independently. Elements with concentrations of 1 μM or higher are shown in the fig. *P*-values were calculated by two-tailed Welch’s *t*-test for Zn and two-tailed Wilcoxon rank-sum test for other components. (C) Blastocyst formation rate in culture medium supplemented with ZnCl_2_ or B(OH)_3_. Each dot represents the blastocyst formation rate of 12 embryos. The lines and the number of dots indicate the logistic curves and replicates, respectively. The experiments were repeated three times independently.

To explore the mechanism underlying glass toxicity, KSOM^AA^ medium was added to glass-bottom dishes, and the dishes were incubated at 37°C, 6% CO_2_, and 5% O_2_ for 4 days. UPLC-MS/MS and IC were used to analyze the changes in the levels of KSOM^AA^ components, except for BSA, before and after incubation. The results showed that there were no significant differences in the contents of the 28 components with or without glass, except for pyruvate and Cys (Figure 2A). However, the differences in the contents of pyruvate and Cys were 2.8% and 3.6%, respectively. These data suggested that toxicity was not caused by the adsorption or denaturation of the medium components by the glass but by the leaching of toxic substances from the glass. ICP-MS analysis of the trace elements eluted from the glass-bottom dish and glass filter revealed that B, Ti, Zn, Br, and Ba were eluted (Figure 2B). Among these trace elements, B (0–500 μM) and Zn (5–80 μM) were highly exuded by the toxic dish (Figure 1B, company F) as well as the filter (Figure 1A, company B). To determine which of these elements was more toxic for embryonic development, boronic acid and zinc chloride were added to the culture medium of fertilized mouse eggs, and the blastocyst development rate was examined. Boronic acid was not toxic even at a concentration of 40 μM (approximately five times the concentration detected in the dish, Figure 2B), whereas ZnCl_2_ significantly inhibited embryonic development at 6 μM (Figure 2C). Zn from the glass-bottom dish leached into the culture medium over time, exceeding 3 μM after 1 day of medium equilibration and gradually increased to 6 μM over the next 4 days (Supplemental Figure S1C). The blastocyst development rates in the medium on glass-bottom dishes or in the ZnCl_2_-added medium (3 μM and 6 μM) on plastic dishes were 12.5%, 17.6%, and 10.6%, respectively (Supplemental Figure S1D). The basal Zn concentration in the untreated KSOM^AA^ medium used was consistently low at 0.102 ± 0.045 μM (mean ± SD) in eight batches. This indicated that the observed toxicity of the glass products may be partly attributed to the leaching of Zn into the culture medium.

### Window of sensitivity for the developmental toxicity of Zn

**Figure 3:**
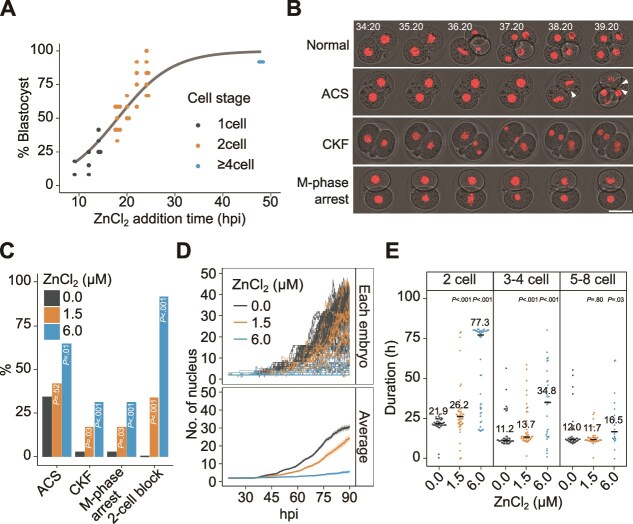
Sensitivity window to Zn and abnormalities in preimplantation mouse embryos. (A) Blastocyst formation rate on Day 4 of pronuclear-stage embryos exposed to 6 μM ZnCl_2_ from 9 to 48 h post-insemination (hpi). Each dot represents the blastocyst formation rate of 11–13 embryos. The line and the number of dots indicate the logistic curve and replicates, respectively. The experiments were repeated thrice independently. (B–E) Observation of abnormalities by live-cell imaging for 4 days of 0.0, 1.5, and 6.0 μM ZnCl_2_-exposed embryos injected with a histone H2B-mCherry probe. In two independent experiments, four replicates of 11–12 embryos in 5 μl of medium per group were conducted (n = 44 for 0.0 μM, n = 48 for 1.5 μM, n = 48 for 6.0 μM). (B) Typical patterns of abnormalities observed by live-cell imaging. ACS, abnormal chromosome segregation; CKF, cytokinesis failure; M-phase arrest, mitotic phase arrest. Scale bar indicates 50 μm. (C) Frequency of abnormalities counted at least once from one-cell- to eight-cell-stage of embryos. *P-*values for comparisons with the 0.0 μM control were calculated by two-tailed Fisher’s exact tests and adjusted using the Holm procedure. (D) The number of nuclei for individual embryos (top) and the mean values ± SEM (bottom) from 20 to 90 hpi. (E) Duration (h) of two-cell, three- to four-cell, and five- to eight-cell stages. The median values are represented by lines and numbers. *P-*values for comparisons with the 0.0 μM control were calculated using the two-tailed Steel test.

To identify the developmental period during which the embryos were sensitive to Zn, we added ZnCl_2_ at a final concentration of 6 μM (equivalent to the concentration of Zn in the medium on a glass-bottom dish) after insemination and observed development until the blastocyst stage. We identified a period of high sensitivity that began immediately after fertilization and lasted until the early two-cell stage (Figure 3A). Mouse embryos derived from inbred or outbred strains, which are associated with increased two-cell block *in vitro* [33], were more sensitive to Zn than those derived from hybrid strains (Supplemental Figure S2). The effects of Zn on chromosome segregation, cytokinesis, and developmental rate were examined using live-cell imaging. The frequency of chromosome misalignment, chromosome lag, and resulting micronucleus formation during chromosome segregation (abnormal chromosome segregation), along with the appearance of multiple nuclei associated with abnormal cytokinesis (cytokinesis failure) increased significantly in a Zn concentration-dependent manner (Figure 3B, C). Moreover, the frequency of embryos exhibiting mitotic phase arrest and two-cell block increased (Figure 3B, C). When we measured the developmental rate by counting the change in the number of nuclei from the two-cell- to blastocyst-stage embryos using our proprietary AI program [21], we observed developmental delay and arrest that increased in a Zn concentration-dependent manner (Figure 3D), especially at the two-cell stage (Figure 3E).

### Embryonic gene expression in response to Zn

**Figure 4:**
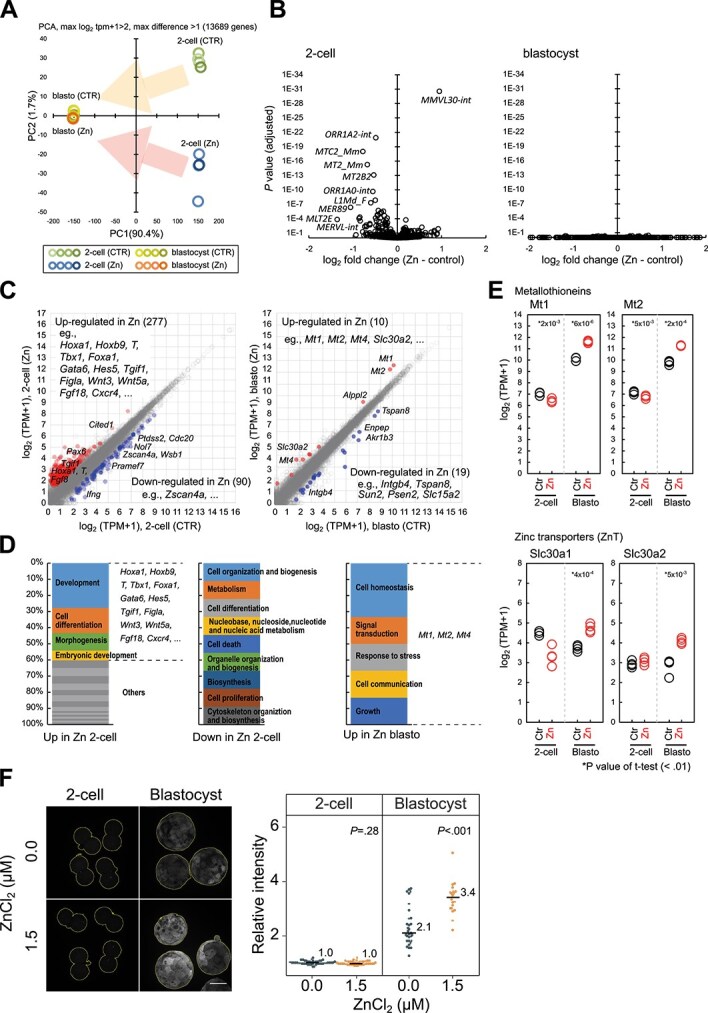
Transcriptome analysis of preimplantation mouse embryos after Zn exposure. (A) Principal component analysis of two-cell embryos and blastocysts with and without 1.5 μM ZnCl_2_. The arrows indicate the change from the two-cell to blastocyst stage in the same group. The cDNA library preparations for transcriptome analysis were replicated four times using independently pooled two-cell-stage embryos (n = 20) and blastocyst-stage embryos (n = 8). (B) Volcano plots for the expression of transposable elements in two-cell embryos (left) and blastocysts (right). Log_2_ fold change between Zn-treated and control embryos (averages of biological replicates) and adjusted *P-*values are plotted; representative transposons are shown. (C) Scatterplots of gene expression (log_2_ TPM + 1) in Zn-treated and control embryos in two-cell and blastocyst stages. The averages of four biological replicates are plotted. Up- and downregulated genes are represented by red and blue open circles, respectively. The representative genes are listed below. (D) Stacked bar graphs of the GO Slim classification for upregulated and downregulated genes in Zn-treated two-cell embryos (left and middle, respectively) and upregulated genes in Zn-treated blastocysts (right). (E) Expression (log_2_ TPM + 1) of individual genes in Zn-treated (red open circles) and control (black open circles) embryos in two-cell and blastocyst stages. *P-*values were calculated using the two-tailed Student’s *t*-test. (F) Immunostaining results for metallothioneins (MT1 and MT2) in two-cell-stage and blastocyst-stage embryos with and without 1.5 μM ZnCl_2_. Scale bar indicates 50 μm (left). Relative fluorescence intensity of immunostaining (two-cell: n = 44 for 0.0 μM, n = 40 for 1.5 μM; blastocyst: n = 31 for 0.0 μM, n = 20 for 1.5 μM) (right). The median values are represented by lines and numbers. *P*-values were calculated using two-tailed Student’s *t*-test for two-cell-stage embryos, and two-tailed Wilcoxon rank-sum test for blastocysts. The experiments were repeated three times independently.

To investigate the transcriptomic impact of Zn exposure on zygotes, we performed RNA-seq analysis of the two-cell-stage embryos and blastocysts. At 6 μM ZnCl_2_, which causes significant growth retardation (Figure 3D, E), it was difficult to assess whether the differentially expressed genes resulted from growth retardation or Zn exposure. Therefore, the embryos were cultured with 1.5 μM ZnCl_2_, which resulted in less developmental delay compared to 6 μM ZnCl_2_ and a higher blastocyst development rate (83.3%) (Figure 3D, E and Supplemental Figure S3). Furthermore, only expanded blastocysts that were morphologically similar to those without ZnCl_2_ were selected (Supplemental Figure S3). Unsupervised hierarchical clustering of single-copy genes (i.e. non-repetitive elements) showed that biological replicates were grouped in the same clusters, indicating the high reproducibility of this assay (Supplemental Figure S4A). PCA revealed that a variety of gene expression profiles were attributed primarily to differences in the developmental stage (two-cell embryos vs. blastocysts; PC1, 90%) and the impact of Zn exposure (PC2, 1.7%) (Figure 4A). The two-cell-stage embryos showed considerable aberration under Zn exposure, whereas the blastocyst-stage embryos showed fewer signs of disturbance (Figure 4A).

Next, we investigated repetitive elements and found that retrotransposons characteristically activated during zygotic gene activation (ZGA) (e.g. *MERVL*, *ORR1A*) were downregulated by the addition of Zn at the two-cell stage, suggesting repression and/or delay of ZGA (Figure 4B). Interestingly, *MMVL30-int* levels increased considerably the Zn treatment. At the blastocyst stage, few significant transposon expression differences were observed between the Zn-treated and control embryos (Figure 4B).

Next, we analyzed individual differentially expressed genes between control and Zn-treated embryos and found that 277 and 90 genes were up- and downregulated in the Zn group compared with the control group, respectively, in the two-cell stage (Figure 4C). The majority of the upregulated genes (e.g. *Hoxa1, Hoxb9, T,* and *Fgf8*) were barely detectable during the normal preimplantation stage (Supplemental Figure S5) and were enriched for developmental regulators (Figure 4D). The downregulated genes included those specific to ZGA [34, 35], including *Zscan4a*, a paralog of *Zscan4c*, which is essential for the maintenance of genomic integrity [36, 37] and the activation of *MERVL* [38].

In contrast, blastocysts showed only 10 and 19 up- and downregulated genes in the Zn group compared with the control group, respectively (Figure 4C). The upregulated genes were highly enriched in relation to factors involved in Zn metabolism, including Zn exporters (*Slc30a1/ZnT1* and *Slc30a2/ZnT2*) and metallothioneins (*Mt1* and *Mt2*) (Figure 4E and Supplemental Figure S4B). Immunostaining revealed that MT proteins were upregulated in the blastocyst trophectoderm (Figure 4F and Supplemental Figure S4C). At the two-cell stage, the expression of these Zn buffering factors was much lower than that at the blastocyst stage (Figure 4E) and was not upregulated by Zn exposure (Figure 4E). These factors are upregulated during normal development [29] (Supplemental Figure S6) and may underlie the Zn tolerance of the blastocysts.

### Candidate transcription factors for Zn-induced gene expression abnormalities

To obtain mechanistic insights into the observed Zn-induced expression changes, we explored the transcription factor–binding motifs in the promoter sequences (within 1 Kbp of the transcription start site) of the differentially expressed genes. We found that the genes upregulated by Zn at the two-cell stage were strongly enriched with binding motifs of Zn-finger transcription factors, such as metal regulatory transcription factor 1 (MTF1) (*P* < 10^−15^) (Supplemental Figure S7A). The expression of *Mtf1* itself was not altered by Zn at the two-cell or blastocyst stage (Supplemental Figure S7B).

Taken together, our data suggest that Zn exposure perturbed ZGA and induced aberrant upregulation of later developmental programs through Zn-mediated transcription factors, leading to the development of Zn tolerance during preimplantation.

### Effects of preimplantation Zn exposure on post-implantation development

**Figure 5:**
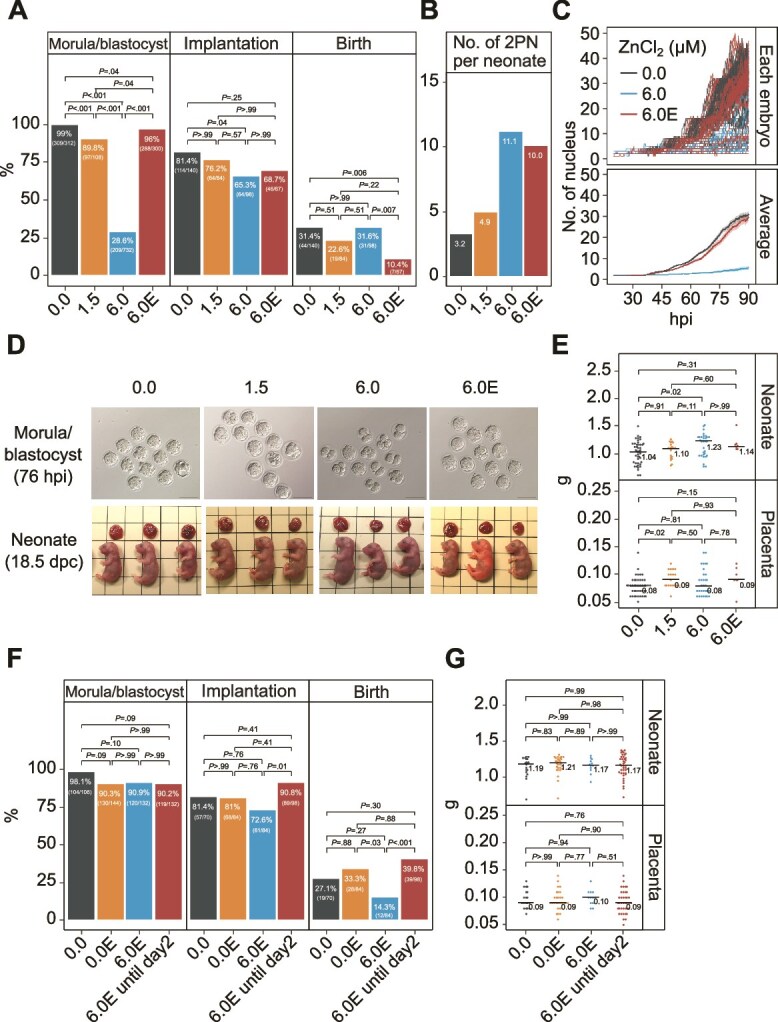
Effects of Zn exposure and chelation of Zn with EDTA on post-implantation mouse development. Pronuclear-stage embryos were cultured in 5 μl of medium supplemented with or without ZnCl_2_ and EDTA until 76 h after insemination. Embryos were transferred to recipient mice 2.5 d post-coitum (dpc) and Cesarean sections were performed at 18.5 dpc. The groups 0.0, 1.5, 6.0, 0.0E, 6.0E, and 6.0E until Day 2 indicate 0.0 μM ZnCl_2_, 1.5 μM ZnCl_2_, 6.0 μM ZnCl_2_, 0.0 μM ZnCl_2_ plus 100 μM EDTA, 6.0 μM ZnCl_2_ plus 100 μM EDTA, and medium change from 6.0E to 0.0 at Day 2, respectively. (A, F) Morula or blastocyst formation rate (Morula or blastocyst, left), implantation rate (middle), and birth rate (right). The experiments were replicated four times (A) and twice (F) independently. *P*-values were calculated using two-tailed Fisher’s exact test and adjusted using the Holm procedure. (B) The number of two pronuclear (2PN) embryos required to obtain one neonate. (C) The number of nuclei for individual embryos (top) and the mean values ± SEM (bottom) from 20 to 90 hpi. In two independent experiments, four replicates of 11–12 embryos in 5 μl of medium per group were conducted (n = 44 for 0.0, n = 48 for 6.0, n = 47 for 6.0E). (D) Photographs of embryos cultured with 0.0, 1.5, 6.0 μM ZnCl_2_ and 6.0E at 76 hpi. Scale bars indicate 100 μm (top). Photographs of neonates at 18.5 dpc after embryo transfer of the embryos into pseudopregnant mice. One side of a square indicates 1 mm (bottom). (E, G) Weight of neonate and placenta (sample size is shown in the bar graph in A and F). *P-*values were calculated using the two-tailed Steel–Dwass test.

We examined the effects of Zn exposure during blastocyst culture on subsequent full-term development. When desirable morula or blastocysts were selected and transferred to pseudopregnant female mice, the birth rate of Zn-treated embryos was comparable to that of control embryos (Figure 5A). However, the morula or blastocyst formation rate in culture decreased in a Zn concentration-dependent manner (Figure 5A, D), such that the number of fertilized eggs necessary to obtain one neonate was significantly higher in the Zn-treated groups than in the control group (Figure 5B). These data suggest that Zn treatment at the preimplantation stage had a strong impact on the formation of the morula or blastocyst but not on post-implantation. Neonates derived from the Zn-treated embryos showed a Zn concentration-dependent increase in birth weight compared with those from control mice (~6% and ~18% higher weights under 1.5 and 6.0 μM ZnCl_2_, respectively; Figure 5E).

### Antagonists of Zn toxicity

Because Zn is a divalent cation, we aimed to cancel its toxicity by adding EDTA, a chelating agent, to the culture medium. KSOM^AA^ medium contains 10 μM EDTA; however, this concentration was clearly insufficient to offset the toxicity of Zn (Figure 5A, D). Increasing the EDTA concentration to 100 μM in 6 μM Zn-supplemented medium dramatically increased the morula or blastocyst formation rate compared with 10 μM EDTA (Figure 5A, 5D), with a developmental speed comparable to that of the control (Figure 5C). The addition of EDTA dramatically improved the blastocyst development rate when toxic syringe filters and glass-bottom dishes were used for culture (Supplemental Figure S8A, B). However, when blastocysts cultured in medium containing 6 μM ZnCl_2_ and 100 μM EDTA were transferred, the birth rate per transferred embryo was lower than that in the control medium (Figure 5B).

Next, we treated the embryos with EDTA only during the Zn sensitive time window (until the 4–8 cell stage; Figure 3A) and observed that the morula or blastocyst development rate, birth rate, placental weight, and birth weight values were comparable to those of the control group (Figure 5F, G). Supplementation with BSA, which is known to have Zn chelating activity [39], improved the blastocyst development rate in the medium discharged from the glass capillary (Supplemental Figure S9A) and the developmental delay in the medium supplemented with Zn (Supplemental Figure S9B). These findings indicated that supplementation with EDTA and/or BSA at an appropriate time during preimplantation development may protect embryos against Zn toxicity, allowing for normal full-term development.

### Zn toxicity in other species

**Figure 6:**
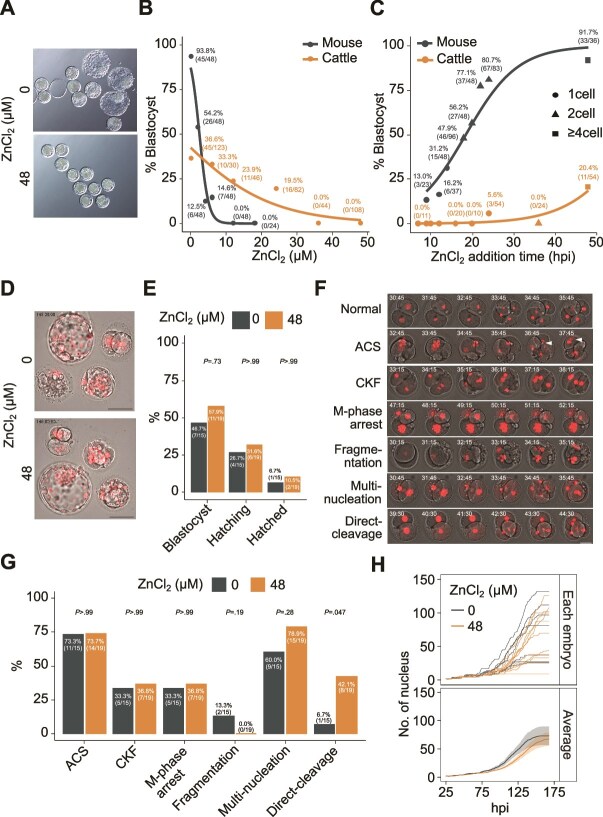
Effects of Zn exposure on bovine and human preimplantation embryos. (A) Photographs of bovine embryos cultured for 8 days with 0 or 48 μM ZnCl_2_ from the one-cell stage. Scale bars indicate 100 μm. (B, C) Comparison of the ZnCl_2_ concentrations (added in the one-cell stage) (B) and the addition times (ZnCl_2_ concentrations in mouse and bovine embryos were 6 and 48 μM, respectively) (C) on mouse and bovine preimplantation embryos. The data for the mice represent the sum of the dataset in Figure 2C and Figure 3A. The bovine embryo experiments were replicated 2–7 times per ZnCl_2_ concentration (B) and 2–3 times per time (C) independently. Lines indicate logistic curves. (D) Images of human embryos cultured for 6 days with 0 or 48 μM ZnCl_2_ from one-cell or two-cell stage. Scale bars indicate 100 μm. (E) Blastocyst, hatching, and hatched rates of human embryos cultured with 48 μM ZnCl_2_ from one-cell or two-cell stage. Thirty-four one-cell or two-cell stage thawed embryos derived from four patients were mixed and randomly allocated to two groups (n = 15 for 0 μM, n = 19 for 48 μM). *P-*values were calculated using the two-tailed Fisher’s exact test. (F) Typical patterns of abnormalities in human embryos injected with a histone H2B-mCherry probe during live-cell imaging. ACS, abnormal chromosome segregation; CKF, cytokinesis failure; M-phase arrest, mitotic phase arrest; fragmentation, fragmentation of blastomeres; multi-nucleation, blastomere with ≥2 nuclei; direct cleavage, multiple cytoplasmic divisions per cleavage. Scale bar indicates 50 μm. (G) Frequency of abnormalities counted at least once from one-cell to eight-cell stage of the embryos. *P-*values were calculated using two-tailed Fisher’s exact test. (H) Number of nuclei in human embryos reaching blastocysts cultured with 0 and 48 μM ZnCl_2_ from 25 to 175 h post-insemination (n = 7 for 0 μM, n = 11 for 48 μM). The number of nuclei for individual embryos (top) and the mean values ± SEM (bottom) are shown. *P*-values between 0 and 48 μM Zn at any time point were calculated using the two tailed Wilcoxon rank-sum test.

Finally, we examined the toxicity of Zn in the embryos of other animal species. Zn was added at various concentrations to bovine embryos at 9–10 h post-insemination and incubated for 8 days. We found that Zn exhibited a dose-dependent negative effect on bovine blastocyst formation (Figure 6A, B). In contrast, the Zn sensitivity of bovine embryos was more moderate than that of mice, with a pronounced developmental defect occurring at concentrations >30 μM and a slightly later period of sensitivity (Figure 6C). This toxicity was eliminated when the embryos were cultured in CR1aa medium [40] containing higher concentrations of BSA and calf serum than IVD101 medium [41] (Supplemental Figure S9C).

We injected histone H2B-mCherry mRNA [42] into surplus human embryos at the late pronuclear stage and observed their development to the blastocyst stage in the presence of ZnCl_2_ via live-cell imaging. Using surplus human embryos, inevitably, lead to delay in the timing of Zn addition in terms of the developmental stage compared with the mouse and bovine experiments. Human embryos cultured with 48 μM ZnCl_2_ showed no significant differences from the controls in terms of microscopic morphology, blastocyst formation rate, or several cleavage parameters, including chromosome segregation and cytoplasmic division (Figure 6D–G). The progress of development appeared to be slightly delayed in the Zn-treated embryos; however, the difference was not significant based on the Wilcoxon rank-sum test (Figure 6H). Moreover, the occurrence of direct cleavage, a type of aberrant blastomere cleavage characterized by multiple cytoplasmic divisions per cleavage, was significantly higher in the Zn-treated embryos (42%, 8/19) than in the controls (7%, 1/15). These results suggest that the Zn exuded from the glass exhibited a non-negligible effect on embryonic development in bovine and human embryos, although the degree and time window of sensitivity to Zn differed between the species.

## Discussion

In the present study, we found that Zn leached from the glassware used in embryo culture adversely affects embryo development. Zn is the second most abundant trace element after iron and is essential for all living organisms. It binds to ~10% of all proteins in the human body (~3000 proteins) [43] and is involved in maintaining protein structure, regulating enzyme activity, and acting as a signal transducer inside and outside cells [44]. Recent studies have also reported that Zn plays a critical role in the normal progression of oocyte growth and maturation, fertilization, epigenetic programming, and subsequent embryonic, fetal, and placental development, and that an excess or deficiency of Zn can cause various abnormalities [45]. In human blood, the total Zn concentration is 10–25 μM [46]; however, biologically active, i.e. free Zn, concentrations are in the pM range [47] because most of the Zn is bound to carrier proteins, such as albumin and α2-macroglobulin, which constitute ~5% and 0.2% of the blood, respectively [48]. The concentration of Zn in the uterine fluid that supports the development of preimplantation embryos is 2.4–49.3 μM [49, 50]. The presence of albumin in the female reproductive tract has been confirmed [51–53], suggesting that the free Zn concentration is maintained at low levels to avoid adverse effects on the embryo *in vivo*. However, *in vitro*, the carrier proteins of Zn are either absent or present at low levels in the embryo culture media (α2-macroglobulin is absent and the albumin levels are 0.1%–0.5%) [4]. EDTA, a chelator of divalent cations, has sometimes been added to culture medium to inhibit the adverse effects of Fe and Zn on embryo development [13, 54] since Abramczuk et al. found it effective in alleviating two-cell block in 1977 [33]. In the present study, we primarily used KSOM^AA^ medium containing 0.1% BSA and 10 μM EDTA, which has been used extensively to culture embryos; however, Zn-binding agents were not present at sufficient levels to cancel the toxicity of Zn exuded from glassware in some cases.

The Zn concentration in the culture medium increases with the time of contact with the glassware. For example, the Zn concentration in the culture medium on the glass-bottom dishes was >3 μM at the beginning of the culture and then increased to 6 μM over a period of 4 days (Supplemental Figure S1C). The contact area with the glassware is also an important factor. Increasing the contact area of the medium on the cover glass decreased the blastocyst development rate (Supplemental Figure S1B). The higher amount of Zn leached from the filter than from the glass-bottom dish is attributed to the fibrous nature of glass inside the filter, which had a larger contact area. Harrison et al. showed that filters used in liquid sterilization are embryotoxic and attributed this to the residual ethylene oxide used for sterilization and the quality of the plastic casings; however, the present results also suggest the involvement of Zn [12]. Thus, the contact time and area between the embryo culture medium and glassware should be minimized. Zn may enter the culture medium from sources other than glassware and interfere with embryonic development. Erbach et al. showed that Zn in the silicone oil used in microdrop culture could enter the medium and interfere with the development of mouse preimplantation embryos [13]. In this report, a Zn concentration of 27 μM was estimated to inhibit the development of 50% of the mouse embryos into blastocysts when KSOM medium containing 0.1% BSA and 10 μM EDTA was used. Erbach et al reported higher embryotoxic concentration (27 μM) than those in our study (less than 10 μM, Supplemental Figure S2) that can be attributed to the different experimental conditions, such as different sensitivities to Zn in different mouse strains (Supplemental Figure S2) and differences in the developmental potential of *in vitro*-and *in vivo*-fertilized embryos [55, 56]. Nevertheless, exogenous Zn concentrations of a few micromolars to tens of micromolars are considered sufficient to disturb preimplantation embryonic development in mice.

In the present study, ~5–80 μM of Zn was found to be exuded into the culture medium during normal glassware usage. Notably, pronounced toxicity was observed in the mouse embryos with exposure to only 6 μM Zn (Figure 2C). Bovine embryos also exhibited sensitivity to Zn within the range leached from glassware, whereas human embryos appeared to be less sensitive (Figure 6). However, because the human embryos used were vitrified at the latter half of the one-cell stage, Zn was added just before and after the two-cell stage. Specifically, the evaluation of Zn toxicity in human embryos was conducted at a later developmental stage than in the mouse and bovine embryo experiments. Therefore, it is possible that the addition of Zn to human embryos was conducted after the sensitivity period. Because access to human embryos for research is ethically and technically limited, new methodologies, such as *in vitro* egg reconstruction, are required to examine Zn toxicity in detail.

Borosilicate glass, which exhibits chemical resistance and low thermal expansion, is generally used in glassware for research. Many beakers and flasks made of Pyrex® and Duran® are composed of Si, B, Al, and Na but not Zn. Borosilicate glasses used for pharmaceutical research have added Zn to increase their chemical resistance [57, 58]. These Zn-containing glasses are known to leach Zn into the solution [57]. Although the elements of the glass we used are not disclosed, it is possible that Zn was added during glass manufacture. To avoid the risk of toxicity, trace Zn contamination must be investigated and controlled. In bovine embryos, Wooldridge et al. [59] reported that very low concentrations of Zn increased the inner cell mass and total cell number in blastocysts, whereas high Zn levels were embryotoxic. As complete removal of Zn from the culture medium may be inappropriate or unfeasible for preimplantation *in vitro*. Thus, further species-dependent research is needed to determine the ideal Zn concentration and timing of addition to embryo culture. To avoid the negative effects of elevated Zn on pre- and post-embryonic development, additional chelating agents, such as EDTA (Figure 5), albumin, or serum (Supplemental Figure S9), should be added to the culture medium or the glassware should be pre-washed with the culture medium (Figure 1, Supplemental Figure S8C). However, the type, concentration, and timing of exposure to chelating agents that affect embryo development [60] should be considered in detail. Matsukawa et al. reported that four of the seven transition metal chelators (nitrilotriacetate, ethyleneglycolbistetraacetate, dipicolinic acid, and deferoxamine) were less capable of supporting mouse embryonic development [60]. They also reported that the mouse blastocyst development rate reduced significantly at 1000 μM EDTA compared with that at 10–100 μM EDTA. Transition metal chelators form complexes with Zn as well as with Cu, Fe, Ca, and Mg [60]. Excessive quantities of chelating agents may inhibit the function of these elements and negatively affect the embryos. Particularly, membrane-permeable chelating agents such as N,N,N′,N′-tetrakis (2-pyridylmethyl)ethylenediamine (TPEN), which directly interacts with intracellular elements, significantly inhibits mouse embryonic development at low concentrations (1 μM) [61]. EDTA at a concentration of 10–100 μM would be the first choice as a regulator of Zn from the culture environment because it has been used in embryo culture for a long time [33, 62] and has already been reported to attenuate the embryotoxicity of Zn from the silicone oil used in microdrop cultures [13]. However, Lane and Gardner [63] recommended that EDTA should be used only at the cleavage stages because culturing mouse embryos from the cleavage to blastocyst stages in media containing 100 μM EDTA reduces the fetal development rate [64]. We obtained similar results when mouse embryos were cultured in a medium containing 100 μM EDTA and 6 μM Zn (Figure 5A). We had hypothesized that EDTA was responsible for the decrease birth rate; however, the decrease was only observed for the combination of 6 μM Zn and 100 μM EDTA and not for 100 μM EDTA alone (Figure 5F). In somatic cells, Zn-chelator complexes may be more toxic than free Zn [65, 66]. Because EDTA can solubilize metal ions [66], it may act as a Zn reservoir and continue to supply Zn to the embryos over time. In the presence of Zn and EDTA, a decrease in the birth rate was observed despite a high morula or blastocyst formation rate (96%, Figure 5A) and a normal developmental speed (Figure 5C). This indicates that the current culture media may contain risk factors that negatively affect peri- and post-implantation embryos but not preimplantation embryos, and that current preimplantation morphokinetic assessments may be ineffective. Thus, to improve the safety and efficacy of ART, it will be necessary to elucidate the effects of Zn and chelating agents on peri- and post-implantation embryos and develop methods to control and monitor them.

Gene expression in mouse embryos showed greater variation at the two-cell stage than at the blastocyst stage when exposed to low Zn levels. A motif search of the promoter sequences of genes showing altered expression revealed that more than half of the differentially expressed genes included a binding sequence for MTF1 (*P* < 10^−15^) (Supplemental Figure S7). MTF1 is a well-known Zn-finger transcription factor that binds to metal-responsive elements in a Zn-dependent manner. Its target genes include *Mt1*, *Mt2*, and Zn transporter genes [67–69]. Other Zn-finger transcription factor–binding sites were also enriched; albeit, with relatively low *P-*values. This suggests that abnormal gene expression at the two-cell stage resulted from extracellular Zn disrupting intracellular Zn homeostasis and the DNA-binding activity of Zn-finger transcription factors [70]. The PCA results showed that the gene expression of blastocysts that met the morphological criteria for embryo transfer was not distinctly different from that of control embryos; although, the 2-cell-stage embryos showed gene expression changes upon Zn addition. The birth rate of these blastocysts was similar to that of the control, and because >70% of the blastocysts in the 1.5 μM Zn treatment group met the morphological criteria for embryo transfer (Supplemental Figure S3), the altered gene expression induced by Zn at the two-cell stage was likely corrected in the blastocyst stage. Furthermore, the upregulation of *Mt1* and *Mt2* at the blastocyst stage suggested that the embryos adapted to the changes in Zn concentration. However, the possibility that the embryos were heterogeneous before the two-cell stage and that only those tolerant to Zn developed at the earlier blastocyst stage cannot be excluded. Nevertheless, embryos may overcome developmental toxicity through natural selection or adaptation. This strategy ensures the survival of the next generation of organisms; therefore, further detailed analyses are warranted.

This current study’s data suggested that Zn exposure downregulated ZGA-related genes (*e.g. MERVL*, *ORR1A*, *Zscan4a*). Kong et al. also showed that the suppression of intracellular Zn in one- to two-cell-stage mouse embryos with TPEN resulted in zygotic gene expression being reduced by >80% than that in the controls [61]. These findings suggest that Zn plays a crucial role in ZGA progression and that an excess or deficiency of Zn disrupts ZGA. The difference in sensitivity to Zn in mouse, bovine, and human embryos in the present study may be related to the fact that the time of major ZGA is at the two-cell stage in mouse embryos, whereas it is at the four- to eight-cell-stage in bovine and human embryos [35]. However, the relationship between the timing of exposure to exogenous Zn and ZGA requires further investigation.

The developmental origins of the health and disease theory [71], which proposes that early life environmental exposures, especially during the *in utero* period, can have a lasting impact on health and increase susceptibility to disease in adulthood, have recently gained traction. During the ART process, gametes or fertilized eggs are removed from the body and exposed to an environment that is completely different from the tubal environment during the initial developmental period. Consequently, ART babies may be born with a different weight from that of babies conceived spontaneously [72]. As individuals born through ART gradually reach adulthood and middle age, the impact of ART on health and disease can be assessed [73–75]. In the present study, pup weights were significantly increased by Zn in the culture media (Figure 5E). This indicates that the effect of Zn immediately after fertilization affects the phenotype at birth. In a cohort study of 476 women, maternal serum Zn levels were positively correlated with the birth weight of babies [76]. Because serum Zn concentrations have been linked to Zn concentrations in the female reproductive tract [77], Zn may act as a regulator of fetal growth in the pre- and post-implantation stages. Future studies are needed to clarify the specific genes that contribute to the long-term effects of Zn exposure.

Although the widespread use of plastic instruments and filter-sterilized culture media has reduced the use of glass instruments by embryologists and researchers compared with the early days of ART, many methods/procedures for gamete and embryo culture use them, including live-cell imaging [16], microfluidic devices [78], and spindle observation using polarized light microscopy [79]. Although the primary user of sterile filters has changed from experimenters to manufacturers of culture media, the risk to embryos from glass filters has not disappeared. Therefore, we hope that this study raises awareness of glass embryotoxicity not only among embryologists and researchers but also among manufacturers of ART-related products to develop safe and effective methods for assisted reproduction. This study focused on Zn leached from glass; however, further research is needed on the effects of other components, such as glass leachates other than Zn and adhesives used to bond glass and plastic, to ensure the safety and efficacy of embryo culture.

## Supplementary Material

Fig_S1_Yao_et_al_ioaf050

Fig_S2_Yao_et_al_ioaf050

Fig_S3_Yao_et_al_ioaf050

Fig_S4_Yao_et_al_ioaf050

Fig_S5_Yao_et_al_ioaf050

Fig_S6_Yao_et_al_ioaf050

Fig_S7_Yao_et_al_ioaf050

Fig_S8_Yao_et_al_ioaf050

Fig_S9_Yao_et_al_ioaf050

Table_S1_Yao_et_al_ioaf050

## Data Availability

RNA-seq data were deposited in the Gene Expression Omnibus under accession number GSE163982. Other data are available from the corresponding author upon reasonable request.
