# Supplementary material for: Zinc eluted from glassware is a risk factor for embryo development in human and animal assisted reproduction
Source: Biol Reprod. 2025 Apr 2;112(6):1054–71. doi: 10.1093/biolre/ioaf050 (PMC12192442; doi:10.1093/biolre/ioaf050)
Supplement: Fig_S1_Yao_et_al_ioaf050 [file fig_s1_yao_et_al_ioaf050.pdf]

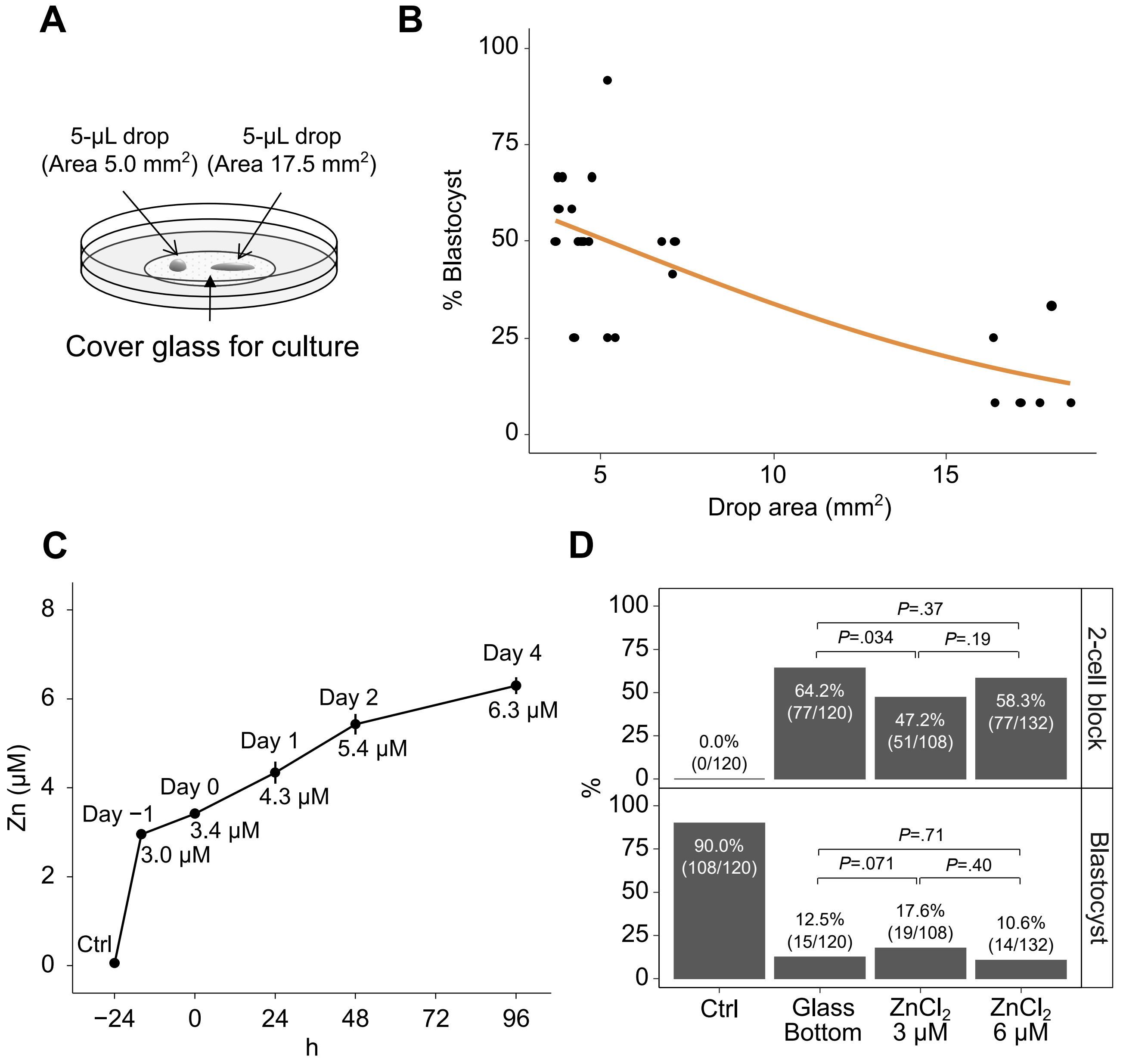

Supplemental Figure S1. Relationship between the contact area and time of culture medium on a cover glass, Zn concentration in the culture medium, and developmental rate of mouse preimplantation embryos.

(A) Pronuclear-stage embryos were cultured for 4 days in 5-μL medium with different contact areas to a cover glass. (B) Relationship between drop area of medium and blastocyst formation rate on day 4. Each dot represents the blastocyst formation rate of 12 embryos in a 5-μL drop. The line and the number of dots indicate the logistic curve and replicates, respectively. The experiments were repeated four times independently. (C) Zn concentrations (the mean ± SEM) in culture medium on glass-bottom dish (Company F) from day −1 (8 hours after the start of equilibration) to day 4. Ctrl, untreated medium before addition to the glass-bottom dish. Sample preparations for Zn analysis were independently replicated thrice using pooled culture media collected from two dishes at each time point. (D) Two-cell block rates and blastocyst formation rates in the medium on glass-bottom dishes or $ZnCl_2$-added medium (3 μM and 6 μM) on plastic dishes. Two-cell block, embryos at the two-cell stage until 48 h post-insemination; Ctrl, culture medium on plastic dish; glass bottom, same batch of dishes analyzed in (C). Experiments were independently replicated twice. *P*-values were calculated using two-tailed Fisher's exact tests and adjusted using the Holm procedure.
