# Supplementary material for: Zinc eluted from glassware is a risk factor for embryo development in human and animal assisted reproduction
Source: Biol Reprod. 2025 Apr 2;112(6):1054–71. doi: 10.1093/biolre/ioaf050 (PMC12192442; doi:10.1093/biolre/ioaf050)
Supplement: Fig_S2_Yao_et_al_ioaf050 [file fig_s2_yao_et_al_ioaf050.pdf]

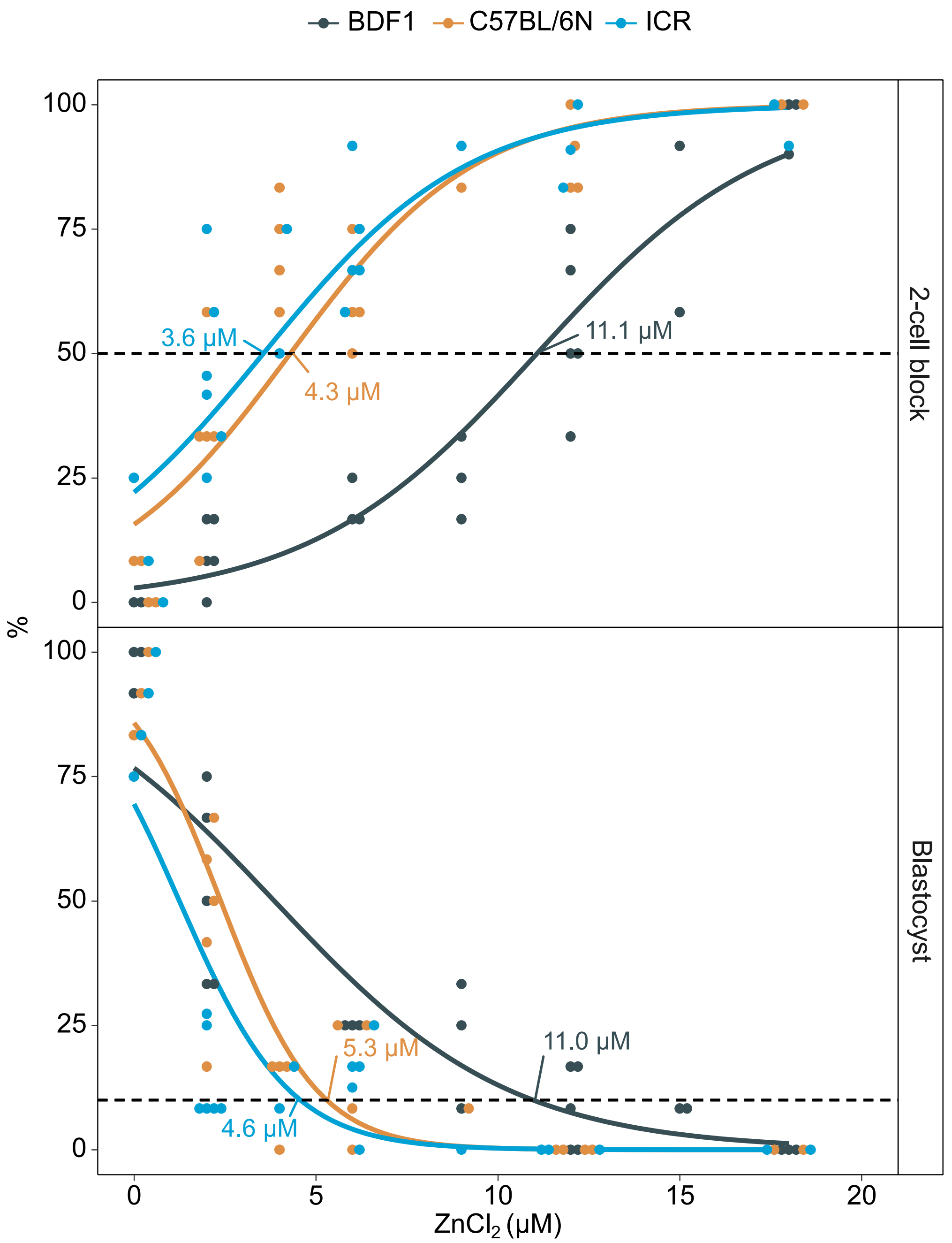

Supplemental Figure S2. Differences in sensitivity to Zn exposure among mouse strains.
Two-cell block and blastocyst formation rates of mouse embryos (BDF1, C57BL/6N, and ICR strains) exposed to various $ZnCl_2$ concentrations from the pronuclear to blastocyst stage for 4 days. Some data for the C57BL6N mice represent the dataset in Fig. 2C. Each dot represents the blastocyst formation rate in 12 embryos. The solid lines and number of dots indicate the logistic curves and replicates, respectively. The experiments were independently repeated thrice. The numeric value with a dashed line indicates the calculated Zn concentration that reduced the two-cell block rate to 50% and the blastocyst formation rate to 10%.
