# Supplementary material for: Zinc eluted from glassware is a risk factor for embryo development in human and animal assisted reproduction
Source: Biol Reprod. 2025 Apr 2;112(6):1054–71. doi: 10.1093/biolre/ioaf050 (PMC12192442; doi:10.1093/biolre/ioaf050)
Supplement: Fig_S3_Yao_et_al_ioaf050 [file fig_s3_yao_et_al_ioaf050.pdf]

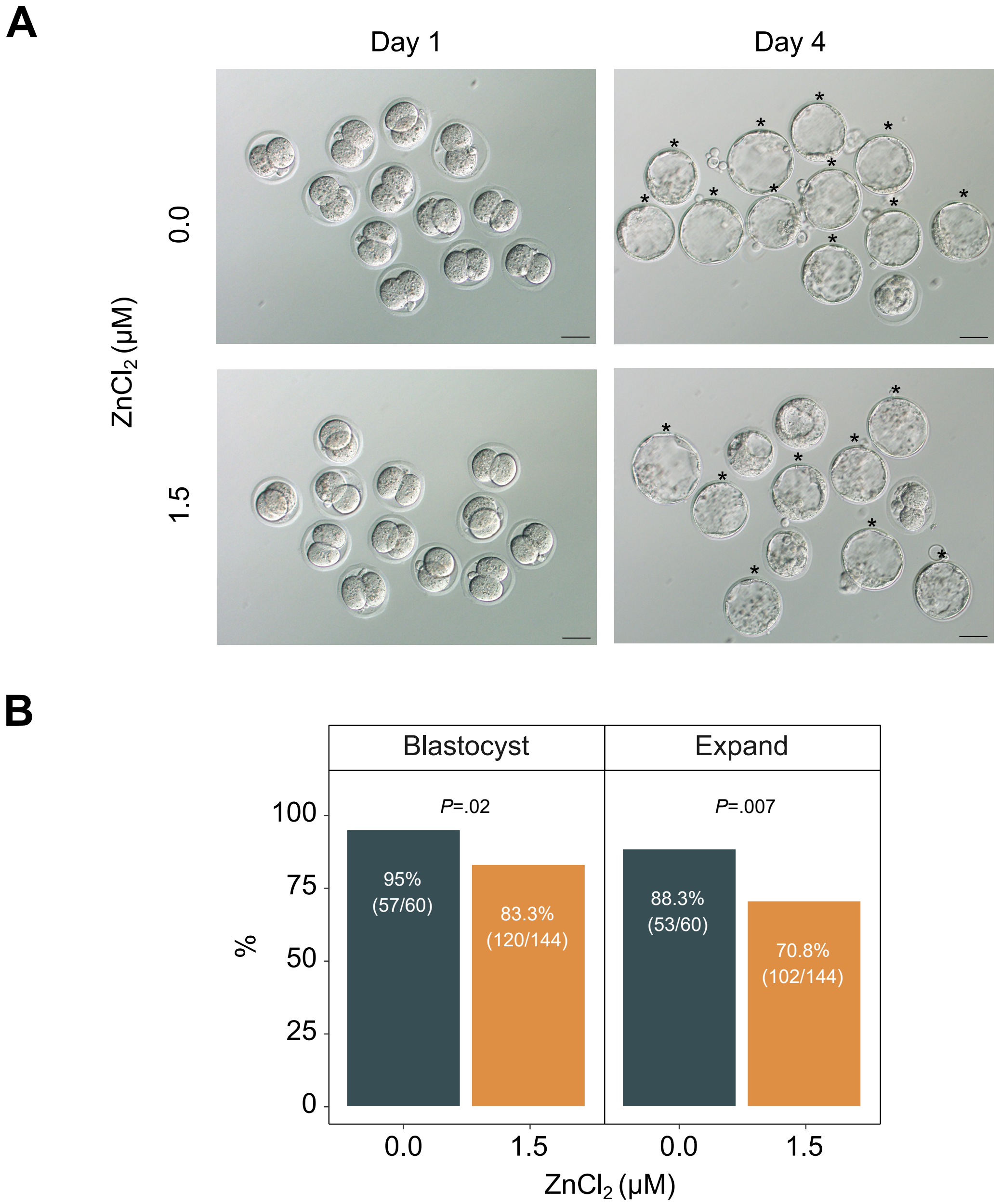


**Supplemental Figure S3. Morphology and developmental rate of mouse embryos used for transcriptome analysis.**

(A) Photographs of embryos cultured with 0.0 and 1.5 μM $ZnCl_2$ on day 1 (left) and 4 (right). Asterisks indicate expanded blastocysts subjected to transcriptome analysis. Scale bars indicate 50 μm. (B) Blastocyst and expanded blastocyst formation rate of embryos cultured with 0.0 and 1.5 μM $ZnCl_2$ on day 4. Five to twelve replicates of 12 embryos in 5-μL medium per group were conducted. *P*-values were calculated using two-tailed Fisher's exact test.
