# Supplementary material for: Zinc eluted from glassware is a risk factor for embryo development in human and animal assisted reproduction
Source: Biol Reprod. 2025 Apr 2;112(6):1054–71. doi: 10.1093/biolre/ioaf050 (PMC12192442; doi:10.1093/biolre/ioaf050)
Supplement: Fig_S4_Yao_et_al_ioaf050 [file fig_s4_yao_et_al_ioaf050.pdf]

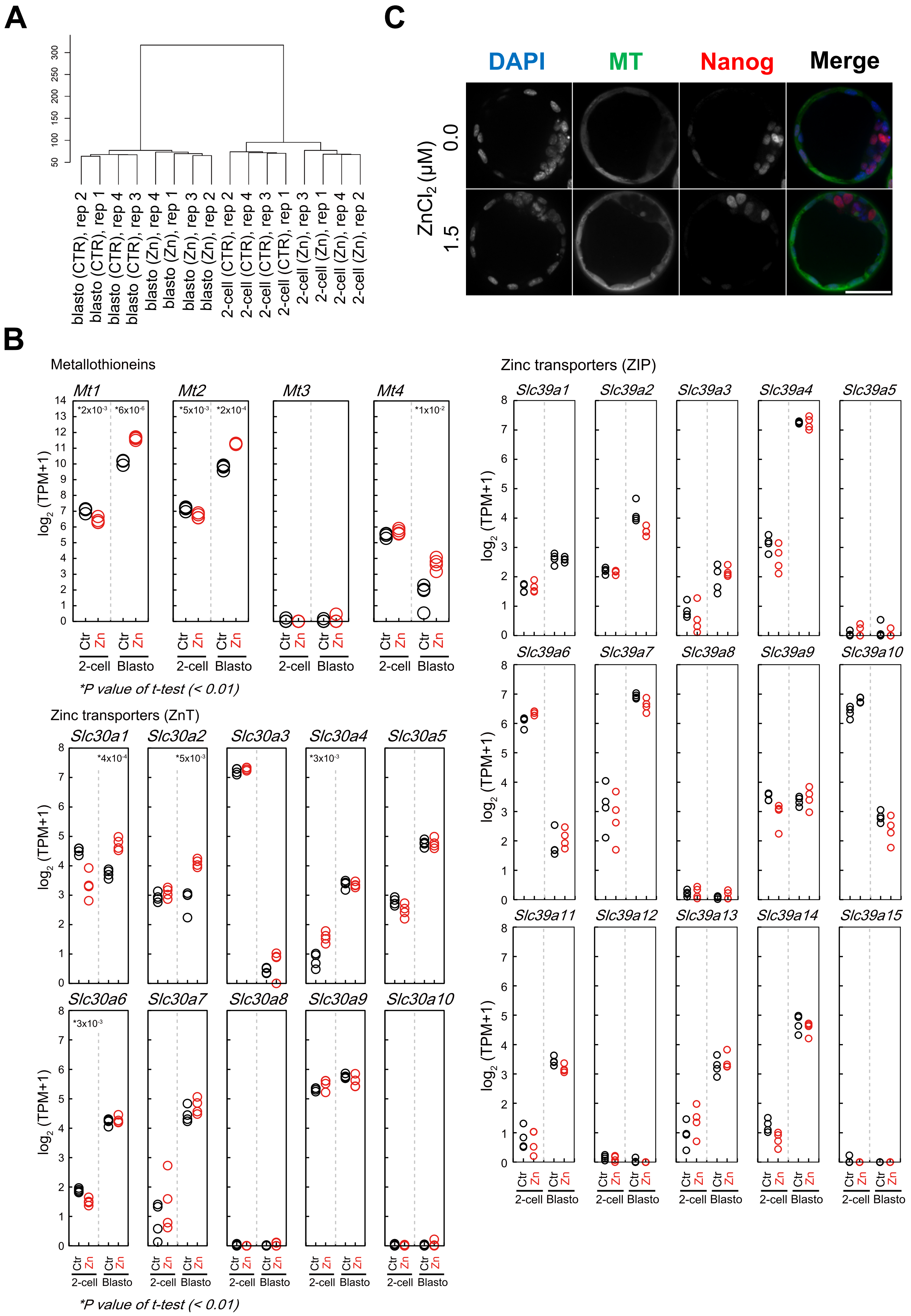

A
blasto (CTR), rep 2
blasto (CTR), rep 1
blasto (CTR), rep 4
blasto (CTR), rep 3
blasto (Zn), rep 4
blasto (Zn), rep 1
blasto (Zn), rep 3
blasto (Zn), rep 2
2-cell (CTR), rep 2
2-cell (CTR), rep 4
2-cell (CTR), rep 3
2-cell (CTR), rep 1
2-cell (Zn), rep 3
2-cell (Zn), rep 1
2-cell (Zn), rep 4
2-cell (Zn), rep 2
C
DAPI
MT
Nanog
Merge
ZnCl2 (μM)
0.0
1.5
B
Metallothioneins
Mt1
Mt2
Mt3
Mt4
*2x10-3
*6x10-6
*5x10-3
*2x10-4
*1x10-2
log2 (TPM+1)
Ctr
Zn
2-cell
Blasto
*P value of t-test (< 0.01)
Zinc transporters (ZnT)
Slc30a1
Slc30a2
Slc30a3
Slc30a4
Slc30a5
*4x10-4
*5x10-3
*3x10-3
Slc30a6
Slc30a7
Slc30a8
Slc30a9
Slc30a10
*3x10-3
*P value of t-test (< 0.01)
Zinc transporters (ZIP)
Slc39a1
Slc39a2
Slc39a3
Slc39a4
Slc39a5
Slc39a6
Slc39a7
Slc39a8
Slc39a9
Slc39a10
Slc39a11
Slc39a12
Slc39a13
Slc39a14
Slc39a15

Supplemental Figure S4. Transcriptome analysis of mouse preimplantation embryos.
(A) Unsupervised hierarchical clustering of nonrepetitive elements. The cDNA library preparations for transcriptome analysis were replicated four times using independently pooled two-cell-stage embryos (n = 20) and blastocyst-stage embryos (n = 8). (B) Expression analysis of individual genes involved in Zn metabolism in control (ctr) and Zn-treated (Zn) 2-cell embryos and blastocysts. Log_2 (TPM + 1) values were plotted. Control and Zn-treated samples are represented by black and red open circles, respectively. *P*-values were calculated using two-tailed Student's *t*-test between the control and Zn-treated embryos. The data shown are from genes encoding metallothioneins (*Mt1*, *Mt2*, *Mt3*, and *Mt4*), ZnT Zn transporters (*Slc30a1*, *Slc30a2*, *Slc30a3*, *Slc30a4*, *Slc30a5*, *Slc30a6*, *Slc30a7*, *Slc30a8*, *Slc30a9*, and *Slc30a10*), and ZIP Zn transporters (*Slc39a1*, *Slc39a2*, *Slc39a3*, *Slc39a4*, *Slc39a5*, *Slc39a6*, *Slc39a7*, *Slc39a8*, *Slc39a9*, *Slc39a10*, *Slc39a11*, *Slc39a12*, *Slc39a13*, *Slc39a14,* and *Slc39a15*). (C) Immunofluorescence with anti-MT and anti-NANOG antibodies and DAPI for mouse blastocysts cultured with 0.0 and 1.5 μM $ZnCl_2$ for 4 days. Scale bar indicates 50 μm.
