# Supplementary material for: Zinc eluted from glassware is a risk factor for embryo development in human and animal assisted reproduction
Source: Biol Reprod. 2025 Apr 2;112(6):1054–71. doi: 10.1093/biolre/ioaf050 (PMC12192442; doi:10.1093/biolre/ioaf050)
Supplement: Fig_S5_Yao_et_al_ioaf050 [file fig_s5_yao_et_al_ioaf050.pdf]

Data from Qiao, Y., Ren, C., Huang, S., Yuan, J., Liu, X., Fan, J., Lin, J., Wu, S., Chen, Q., Bo, X., et al. (2020). High-resolution annotation of the mouse preimplantation embryo transcriptome using long-read sequencing. Nat Commun 11, 2653.

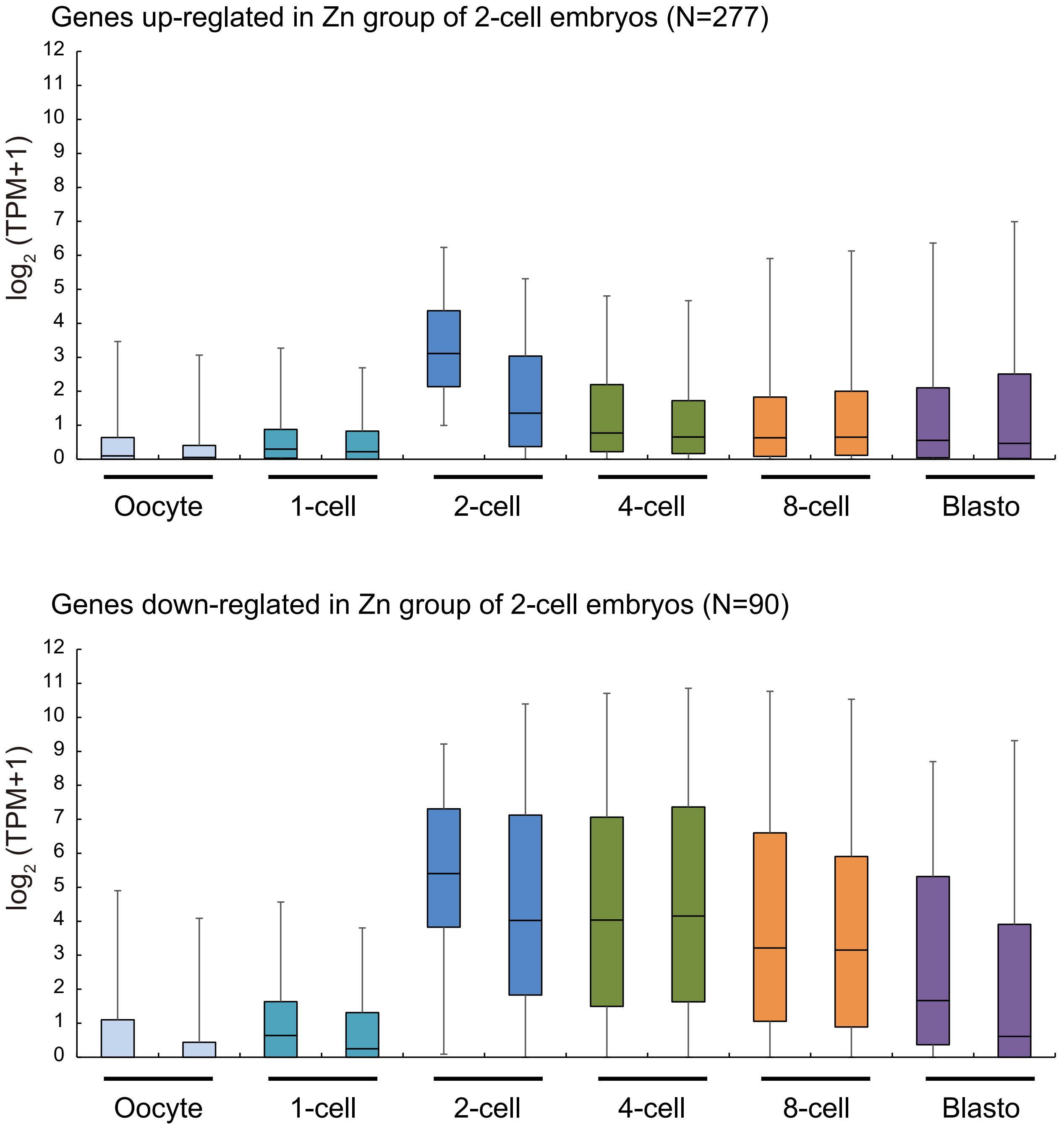

Supplemental Figure S5. Gene expression dynamics in previously published dataset of normal preimplantation development [29].

Genes upregulated and downregulated in Zn-treated 2-cell embryos are shown in the box and whisker plots (277 and 90 genes, respectively). Embryonic stages are indicated by different box colors.
