# Supplementary material for: Zinc eluted from glassware is a risk factor for embryo development in human and animal assisted reproduction
Source: Biol Reprod. 2025 Apr 2;112(6):1054–71. doi: 10.1093/biolre/ioaf050 (PMC12192442; doi:10.1093/biolre/ioaf050)
Supplement: Fig_S6_Yao_et_al_ioaf050 [file fig_s6_yao_et_al_ioaf050.pdf]

Data from Qiao, Y., Ren, C., Huang, S., Yuan, J., Liu, X., Fan, J., Lin, J., Wu, S., Chen, Q., Bo, X., et al. (2020). High-resolution annotation of the mouse preimplantation embryo transcriptome using long-read sequencing. Nat Commun 11, 2653.

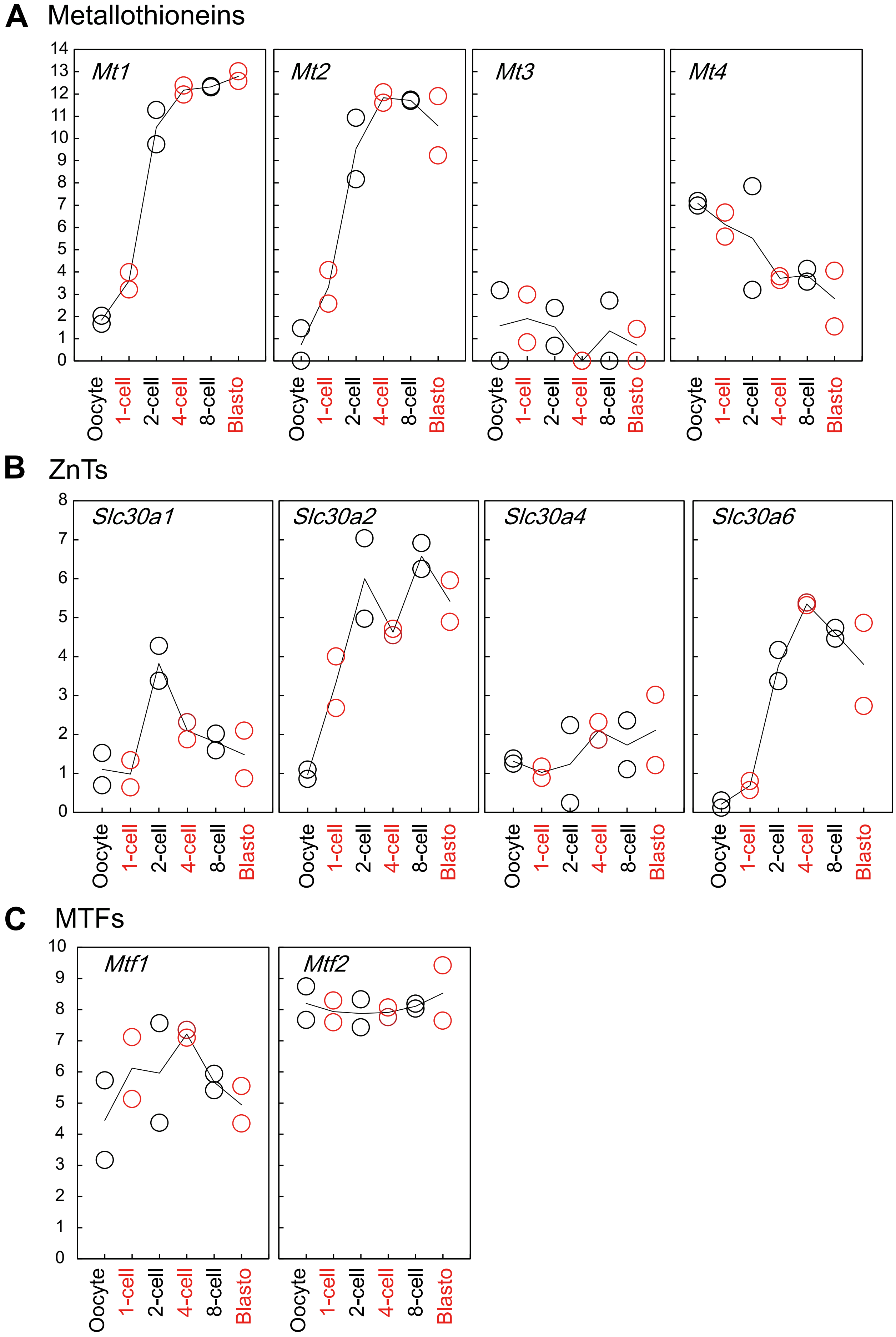

Supplemental Figure S6. Gene expression dynamics in preimplantation mouse embryos using RNA-seq data from a previous study [29].

Log_2 (TPM + 1) values from RNA-seq data [29] are plotted with open circles. The one- and four-cell embryos and blastocysts are colored red. Genes encoding metallothioneins (*Mt1*, *Mt2*, *Mt3*, and *Mt4*) (A), ZnT Zn transporter (*Slc30a1*, *Slc30a2*, *Slc30a3*, and *Slc30a6*) (B), and MTF transcription factors (*Mtf1* and *Mtf2*) (C) are shown.
