# Supplementary material for: Zinc eluted from glassware is a risk factor for embryo development in human and animal assisted reproduction
Source: Biol Reprod. 2025 Apr 2;112(6):1054–71. doi: 10.1093/biolre/ioaf050 (PMC12192442; doi:10.1093/biolre/ioaf050)
Supplement: Fig_S7_Yao_et_al_ioaf050 [file fig_s7_yao_et_al_ioaf050.pdf]

A

Motif enrichment in genomic sequence within 1 kilo-bases from transcription start site (TSS)

Rank	Motif	P-value	log P-pvalue	% of Targets	% of Background	STD(Bg STD)	Best Match/Details
1	TTGCACACGG	1.E-15	-3.7E+01	51.95%	27.41%	535.3bp (691.4bp)	PB0044.1_Mtf1_1/Jaspar(0.908)
2	CTTTGAGCGA	1.E-10	-2.4E+01	12.11%	2.89%	604.9bp (677.8bp)	PB0040.1_Lef1_1/Jaspar(0.665)
3	GGCGCTCATT	1.E-09	-2.3E+01	9.38%	1.81%	337.9bp (571.5bp)	ZNF264(Zf)/HEK293-ZNF264.GFP-ChIP-Seq(GSE58341)/Homer(0.700)
4	CAGCATTTTT	1.E-09	-2.2E+01	10.94%	2.53%	697.0bp (690.0bp)	PB0042.1_Mafk_1/Jaspar(0.787)
5	CTCGAAAGCA	1.E-09	-2.2E+01	52.34%	33.09%	544.8bp (668.0bp)	BCL6B/MA0731.1/Jaspar(0.651)
6	AGCCACTTTG	1.E-09	-2.2E+01	56.64%	37.17%	556.6bp (699.2bp)	Bapx1(Homeobox)/VertebralCol-Bapx1-ChIP-Seq(GSE36672)/Homer(0.713)
7	TTCGTCCCCG	1.E-09	-2.2E+01	13.28%	3.74%	533.3bp (564.3bp)	MZF1/MA0056.1/Jaspar(0.661)
8	TGCTTGCCCC	1.E-09	-2.1E+01	18.75%	7.06%	558.1bp (706.3bp)	Zac1(Zf)/Neuro2A-Plagl1-ChIP-Seq(GSE75942)/Homer(0.670)
9	TGCAGGTGCA	1.E-08	-2.0E+01	16.41%	5.80%	554.1bp (676.8bp)	Slug(Zf)/Mesoderm-Snai2-ChIP-Seq(GSE61475)/Homer(0.809)
10	CCTATCTTTC	1.E-08	-2.0E+01	23.83%	10.73%	583.6bp (710.4bp)	GATA2/MA0036.3/Jaspar(0.654)
11	CCGCATCGAT	1.E-08	-1.9E+01	7.03%	1.19%	464.4bp (568.4bp)	PB0024.1_Gcm1_1/Jaspar(0.699)
12	TGTATACACG	1.E-08	-1.9E+01	7.81%	1.56%	724.2bp (653.0bp)	Foxo3(Forkhead)/U2OS-Foxo3-ChIP-Seq(E-MTAB-2701)/Homer(0.716)
13	TAATGAATCT	1.E-07	-1.8E+01	13.28%	4.35%	551.6bp (696.8bp)	PB0178.1_Sox8_2/Jaspar(0.745)
14	GAGTCGCTTG	1.E-07	-1.8E+01	10.16%	2.73%	554.5bp (600.0bp)	PB0147.1_Max_2/Jaspar(0.651)
15	TGGATGTGAA	1.E-07	-1.7E+01	11.33%	3.45%	528.4bp (705.3bp)	TBX20/MA0689.1/Jaspar(0.712)
16	CTGCTACTCG	1.E-07	-1.6E+01	11.72%	3.78%	450.3bp (613.4bp)	PB0155.1_Osr2_2/Jaspar(0.647)
17	ACCACATCTT	1.E-04	-1.1E+01	5.86%	1.52%	550.5bp (720.2bp)	ETS:RUNX(ETS,Runt)/Jurkat-RUNX1-ChIP-Seq(GSE17954)/Homer(0.728)
18	AAAGGGGGGG	1.E-04	-1.0E+01	23.05%	13.65%	502.9bp (681.0bp)	Maz(Zf)/HepG2-Maz-ChIP-Seq(GSE31477)/Homer(0.720)
19	GGTGTGTC	1.E-04	-9.8E+00	26.56%	16.73%	566.5bp (656.3bp)	KLF10(Zf)/HEK293-KLF10.GFP-ChIP-Seq(GSE58341)/Homer(0.774)
20	ATTCGA	1.E-04	-9.7E+00	39.84%	28.44%	584.6bp (689.8bp)	TEAD3(TEA)/HepG2-TEAD3-ChIP-Seq(Encode)/Homer(0.688)
21	AATACGGA	1.E-04	-9.6E+00	4.30%	0.98%	549.8bp (654.5bp)	PB0106.1_Arid5a_2/Jaspar(0.812)
22	GTGCGA	1.E-03	-9.0E+00	40.23%	29.30%	509.5bp (641.1bp)	MGA/MA0801.1/Jaspar(0.688)
23	TATATATATA	1.E-02	-6.8E+00	5.47%	2.07%	610.2bp (715.9bp)	PB0198.1_Zfp128_2/Jaspar(0.879)

B

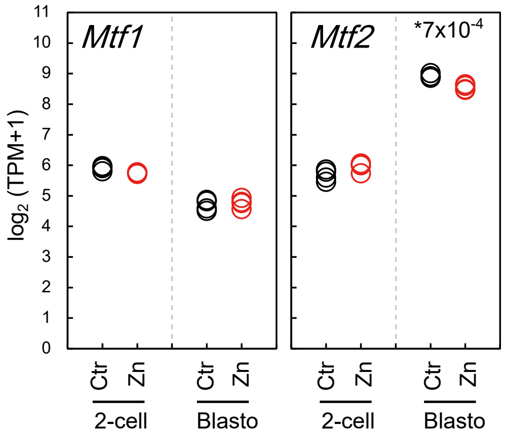

P value of t-test (< 0.01)

Supplemental Figure S7. Motif enrichment analysis for transcription factor–binding sequences.
(A) Genomic sequences around transcription start sites (TSSs) of genes upregulated in Zn-treated two-cell embryos were analyzed using the HOMER program suite, and results of the *de novo* motif search are shown. Sequence logos, *P*-values, frequency of motif-containing sequences (% of targets, % of background), and standard deviation of positions in target and background sequences (STD, Bg STD).
(B) RNA-seq data for MTF transcription factors (*Mtf1* and *Mtf2*).
