# Supplementary material for: Zinc eluted from glassware is a risk factor for embryo development in human and animal assisted reproduction
Source: Biol Reprod. 2025 Apr 2;112(6):1054–71. doi: 10.1093/biolre/ioaf050 (PMC12192442; doi:10.1093/biolre/ioaf050)
Supplement: Fig_S8_Yao_et_al_ioaf050 [file fig_s8_yao_et_al_ioaf050.pdf]

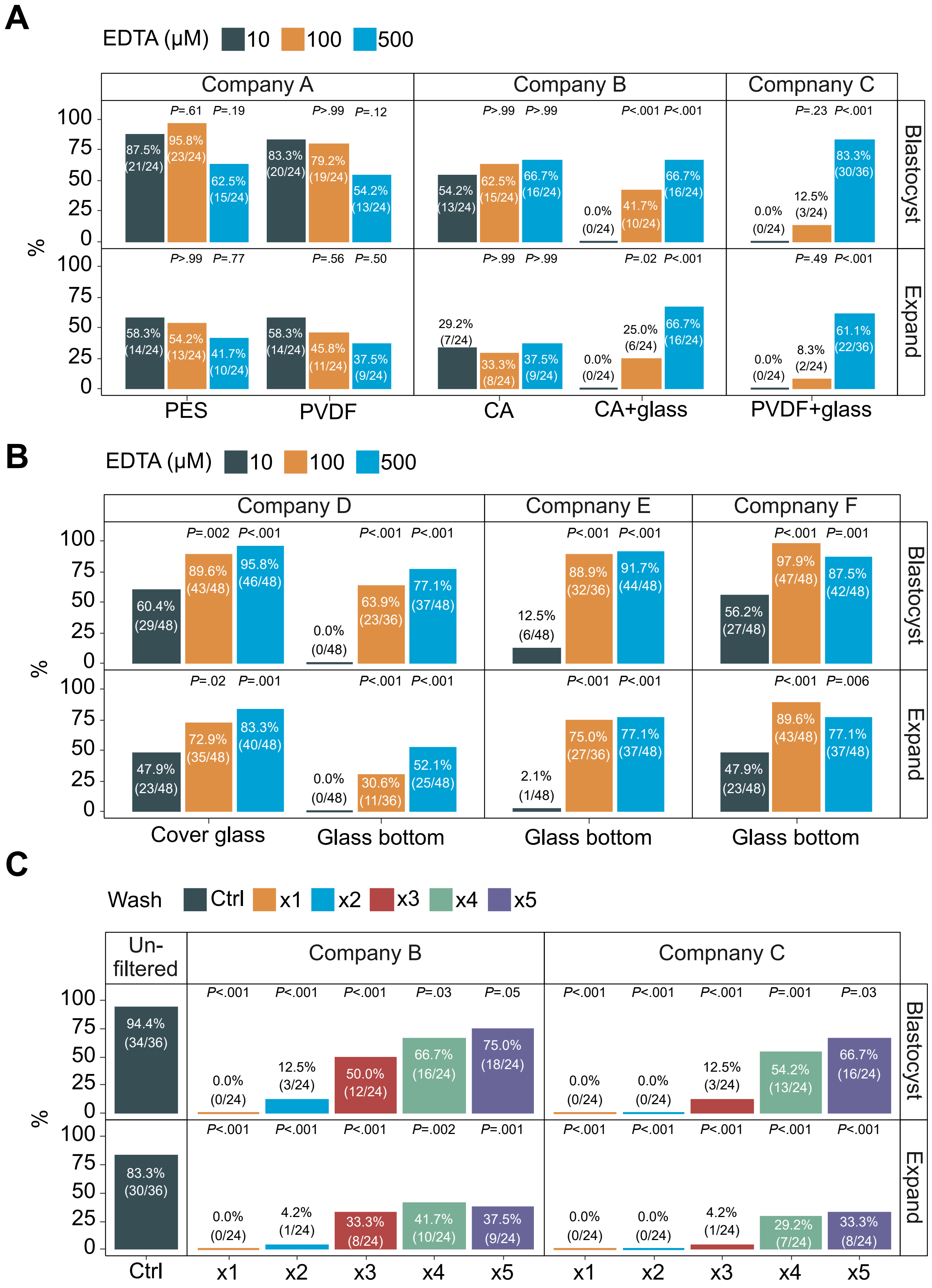
A
EDTA (μM) 10 100 500
Company A
Company B
Compnany C
Blastocyst
Expand
%
100
75
50
25
0
P=.61 P=.19
P>.99 P=.12
P>.99 P>.99
P<.001 P<.001
P=.23 P<.001
87.5% (21/24)
95.8% (23/24)
62.5% (15/24)
83.3% (20/24)
79.2% (19/24)
54.2% (13/24)
54.2% (13/24)
62.5% (15/24)
66.7% (16/24)
0.0% (0/24)
41.7% (10/24)
66.7% (16/24)
0.0% (0/24)
12.5% (3/24)
83.3% (30/36)
P>.99 P=.77
P=.56 P=.50
P>.99 P>.99
P=.02 P<.001
P=.49 P<.001
58.3% (14/24)
54.2% (13/24)
41.7% (10/24)
58.3% (14/24)
45.8% (11/24)
37.5% (9/24)
29.2% (7/24)
33.3% (8/24)
37.5% (9/24)
0.0% (0/24)
25.0% (6/24)
66.7% (16/24)
0.0% (0/24)
8.3% (2/24)
61.1% (22/36)
PES
PVDF
CA
CA+glass
PVDF+glass
B
EDTA (μM) 10 100 500
Company D
Compnany E
Compnany F
Blastocyst
Expand
%
P=.002 P<.001
P<.001 P<.001
P<.001 P<.001
P<.001 P=.001
60.4% (29/48)
89.6% (43/48)
95.8% (46/48)
0.0% (0/48)
63.9% (23/36)
77.1% (37/48)
12.5% (6/48)
88.9% (32/36)
91.7% (44/48)
56.2% (27/48)
97.9% (47/48)
87.5% (42/48)
P=.02 P=.001
P<.001 P<.001
P<.001 P<.001
P<.001 P=.006
47.9% (23/48)
72.9% (35/48)
83.3% (40/48)
0.0% (0/48)
30.6% (11/36)
52.1% (25/48)
2.1% (1/48)
75.0% (27/36)
77.1% (37/48)
47.9% (23/48)
89.6% (43/48)
77.1% (37/48)
Cover glass
Glass bottom
Glass bottom
Glass bottom
C
Wash Ctrl x1 x2 x3 x4 x5
Un-filtered
Company B
Compnany C
Blastocyst
Expand
%
P<.001 P<.001 P<.001 P=.03 P=.05
P<.001 P<.001 P<.001 P=.001 P=.03
94.4% (34/36)
0.0% (0/24)
12.5% (3/24)
50.0% (12/24)
66.7% (16/24)
75.0% (18/24)
0.0% (0/24)
0.0% (0/24)
12.5% (3/24)
54.2% (13/24)
66.7% (16/24)
P<.001 P<.001 P<.001 P=.002 P=.001
P<.001 P<.001 P<.001 P<.001 P<.001
83.3% (30/36)
0.0% (0/24)
4.2% (1/24)
33.3% (8/24)
41.7% (10/24)
37.5% (9/24)
0.0% (0/24)
0.0% (0/24)
4.2% (1/24)
29.2% (7/24)
33.3% (8/24)
Ctrl
x1
x2
x3
x4
x5
x1
x2
x3
x4
x5

Supplemental Figure S8. Preimplantation development of mouse embryos exposed to glassware with chelating agents in culture medium or pre-cleaning of glassware with culture medium.

(A) Blastocyst and expanded blastocyst formation rate of embryos exposed to filtrate collected by passing 1 mL medium through various syringe filters with 10, 100, and 500 μM EDTA. The experiments were replicated twice independently. (B) Blastocyst and expanded blastocyst formation rate of embryos cultured on cover glass or glass-bottom dishes with 10, 100, and 500 μM EDTA. The experiments were replicated twice independently. (C) Blastocyst and expanded blastocyst formation rate of embryos cultured by eluent of pre-washed syringe filters with 1 mL culture medium 1–5 times. The experiments were replicated twice independently. *P*-values were calculated using two-tailed Fisher's exact test and adjusted using the Holm procedure. Twelve embryos in 5-μL medium per group were cultured.
