# Supplementary material for: Zinc eluted from glassware is a risk factor for embryo development in human and animal assisted reproduction
Source: Biol Reprod. 2025 Apr 2;112(6):1054–71. doi: 10.1093/biolre/ioaf050 (PMC12192442; doi:10.1093/biolre/ioaf050)
Supplement: Fig_S9_Yao_et_al_ioaf050 [file fig_s9_yao_et_al_ioaf050.pdf]

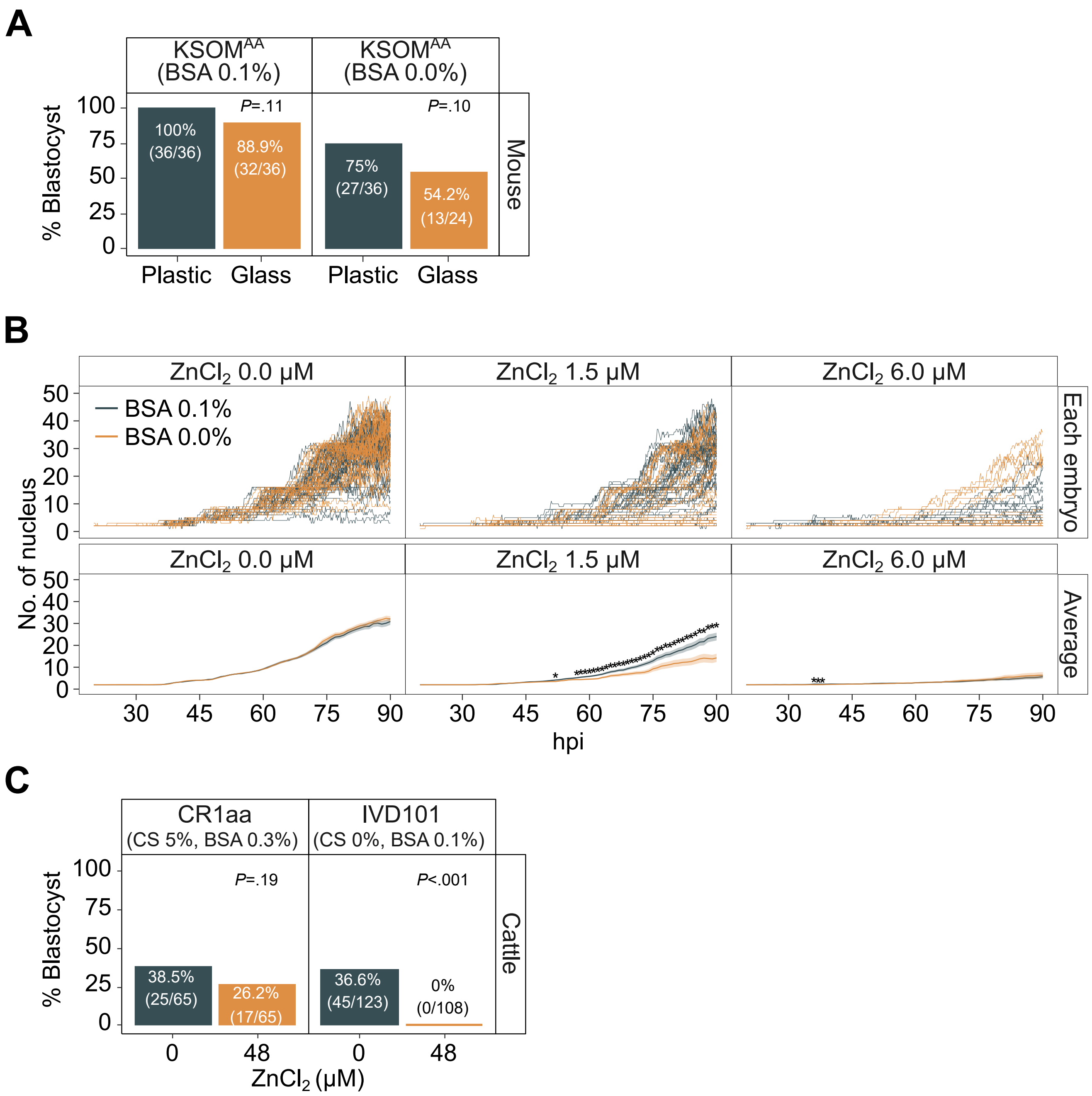

Supplemental Figure S9. Effects of albumin and serum on Zn embryotoxicity.
(A) Blastocyst formation rate of mouse embryos in 5-μL drops of medium with or without BSA discharged from an unwashed plastic or glass capillary. Two to three replicates of 12 embryos in 5-μL medium per group were conducted. *P*-values were calculated using two-tailed Fisher's exact test. (B) Number of nuclei in mouse embryos that were cultured with 0.0, 1.5, or 6.0 μM $ZnCl_2$ with or without BSA during live-cell imaging with histone H2B-mCherry probe from 20 to 90 h post-insemination (hpi). In two independent experiments, four replicates of 11–12 embryos in 5 μL medium per group were conducted (n = 48 for 0.0 μM Zn without BSA, n = 44 for 0.0 μM Zn with BSA, n = 46 for 1.5 μM Zn without BSA, n = 48 for 1.5 μM Zn with BSA, n = 48 for 6.0 μM Zn without BSA, n = 48 for 6.0 μM Zn with BSA). The number of nuclei for individual embryos (top) and the mean values ± SEM (bottom) are shown. Two-tailed Wilcoxon rank-sum test (**P*<.05). (C) Blastocyst formation rate in bovine embryos cultured in serum-supplemented medium (CR1aa) or serum-free medium (IVD101). The experiments were replicated twice for CR1aa and six times for IVD101 independently. *P*-values were calculated using two-tailed Fisher's exact test.
