# Supplementary material for: Zinc eluted from glassware is a risk factor for embryo development in human and animal assisted reproduction
Source: Biol Reprod. 2025 Apr 2;112(6):1054–71. doi: 10.1093/biolre/ioaf050 (PMC12192442; doi:10.1093/biolre/ioaf050)
Supplement: Table_S1_Yao_et_al_ioaf050 [file table_s1_yao_et_al_ioaf050.pdf]

Supplemental Table S1. List of antibodies used in this study.

RRID	Name	Type	Vendor	Catalog number	Dilution in this study
AB_298949	Anti-Metallothionein antibody [UC1MT]	Mouse monoclonal	Abcam	ab12228	1:200
AB_2150114	Anti-Nanog antibody	Rabbit polyclonal	Abcam	ab80892	1:500
AB_10853449	Anti-Mouse IgG (H+L) antibody, CF488A conjugated	Goat polyclonal	Biotium	20018-1	1:500
AB_10854982	Anti-Rabbit IgG (H+L) antibody, CF555 conjugated	Goat polyclonal	Biotium	20232-1	1:500
